# Consolidated bioprocessing of cellulose to itaconic acid by a co-culture of *Trichoderma reesei* and *Ustilago maydis*

**DOI:** 10.1186/s13068-020-01835-4

**Published:** 2020-12-14

**Authors:** Ivan Schlembach, Hamed Hosseinpour Tehrani, Lars M. Blank, Jochen Büchs, Nick Wierckx, Lars Regestein, Miriam A. Rosenbaum

**Affiliations:** 1 Leibniz Institute for Natural Product Research and Infection Biology – Hans-Knöll-Institute, Jena, Germany; 2 Faculty of Biological Sciences, Friedrich-Schiller-University, Jena, Germany; 3 Institute of Applied Microbiology - iAMB, Aachen Biology and Biotechnology - ABBt, RWTH Aachen University, Aachen, Germany; 4 AVT‑Biochemical Engineering, RWTH Aachen University, Aachen, Germany; 5 Institute of Bio- and Geosciences IBG-1: Biotechnology, Forschungszentrum Jülich, Jülich, Germany

**Keywords:** Consolidated bioprocessing, Itaconic acid, Platform chemical, Microbial consortium, Mixed culture, Co-culture, Cellulose, Lignocellulose, Simultaneous saccharification and fermentation, Metabolic engineering

## Abstract

**Background:**

Itaconic acid is a bio-derived platform chemical with uses ranging from polymer synthesis to biofuel production. The efficient conversion of cellulosic waste streams into itaconic acid could thus enable the sustainable production of a variety of substitutes for fossil oil based products. However, the realization of such a process is currently hindered by an expensive conversion of cellulose into fermentable sugars. Here, we present the stepwise development of a fully consolidated bioprocess (CBP), which is capable of directly converting recalcitrant cellulose into itaconic acid without the need for separate cellulose hydrolysis including the application of commercial cellulases. The process is based on a synthetic microbial consortium of the cellulase producer *Trichoderma reesei* and the itaconic acid producing yeast *Ustilago maydis.* A method for process monitoring was developed to estimate cellulose consumption, itaconic acid formation as well as the actual itaconic acid production yield online during co-cultivation.

**Results:**

The efficiency of the process was compared to a simultaneous saccharification and fermentation setup (SSF). Because of the additional substrate consumption of *T. reesei* in the CBP, the itaconic acid yield was significantly lower in the CBP than in the SSF. In order to increase yield and productivity of itaconic acid in the CBP, the population dynamics was manipulated by varying the inoculation delay between *T. reesei* and *U. maydis*. Surprisingly, neither inoculation delay nor inoculation density significantly affected the population development or the CBP performance. Instead, the substrate availability was the most important parameter. *U. maydis* was only able to grow and to produce itaconic acid when the cellulose concentration and thus, the sugar supply rate, was high. Finally, the metabolic processes during fed-batch CBP were analyzed in depth by online respiration measurements. Thereby, substrate availability was again identified as key factor also controlling itaconic acid yield. In summary, an itaconic acid titer of 34 g/L with a total productivity of up to 0.07 g/L/h and a yield of 0.16 g/g could be reached during fed-batch cultivation.

**Conclusion:**

This study demonstrates the feasibility of consortium-based CBP for itaconic acid production and also lays the fundamentals for the development and improvement of similar microbial consortia for cellulose-based organic acid production.

## Introduction

Itaconic acid (IA) is a bio-derived platform chemical with various uses ranging from polymer synthesis to biofuel production. At the current selling price of 1500–1700 USD/ton, itaconic acid is already becoming competitive to replace fossil polyacrylic acid in the production of superabsorbent polymers [1, 2]. Still, to access other bulk markets like methyl methacrylate, which is currently produced from acetone cyanohydrin (about 1000 USD/ton), the price of itaconic acid has to drop further. Besides reducing the processing costs, the price of itaconic acid can be further reduced by using cheaper substrates. For comparison, the current sugar prices are about 310 USD/ton while pulp grade wood costs only about 35 USD/ton [3, 4]. The cheap and sustainable production of itaconic acid from cellulosic waste streams is therefore a highly anticipated goal of current research [5, 6].

**Fig. 1:**
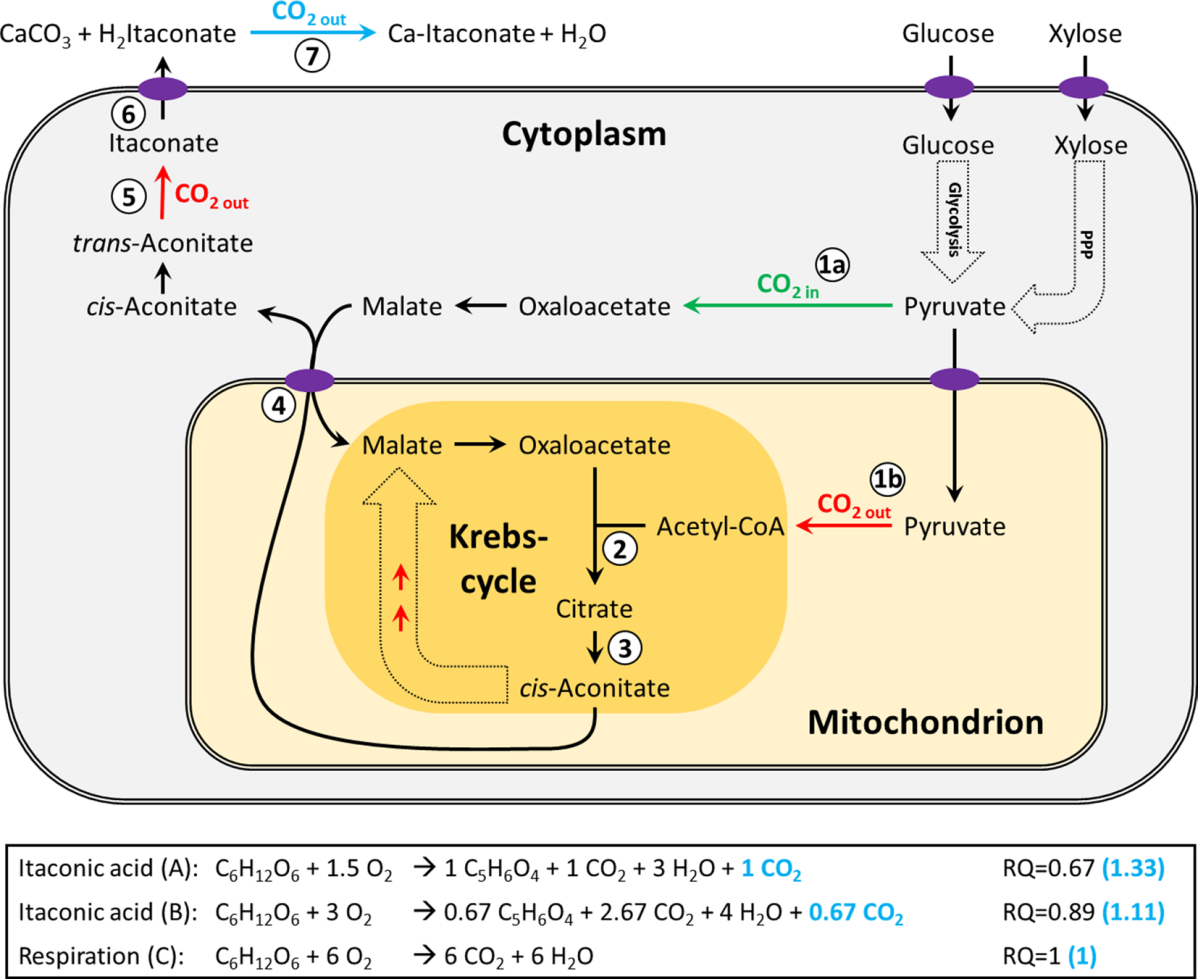
Biosynthesis of itaconic acid. Glucose is taken up from the medium and converted into pyruvate via glycolysis. Pentose sugars are converted to pyruvate via pentose phosphate pathway (PPP). Box shows the reaction stoichiometry and resulting respiratory quotients (RQ) using different pathways: Pathway **a**: To reach the maximum theoretical yield of 1 mol/mol _glucose_, itaconic synthesis must involve anaplerotic CO_2_ fixation: for each mol of glucose, 1 mol of the produced pyruvate is transported to the mitochondrion and 1 mol of pyruvate is converted to malate in the cytosol (1a). In the mitochondrion, pyruvate is converted to acetyl-CoA (1b) and further to citrate by citrate synthase (2) using oxaloacetate. Citrate is then converted into cis-aconitate by aconitase (3). Via a mitochondrial tricarboxylate-malate shuttle (4), cis-aconitate is exported to the cytosol in exchange for malate, which replenishes the oxaloacetate used for citrate synthesis. In the cytosol cis-aconitate is then directly decarboxylated to itaconic acid by cis-aconitate decarboxylase (5) in the case of *A. terreus* or first isomerized into transaconitate before being decarboxylated in the case of *U. maydis*. Finally, itaconic acid is exported outside the cell (6). The respiratory quotient for this pathway would be 0.67. Pathway **b**: If itaconic acid is produced without anaplerotic CO_2_ fixation, the pathway has to be exclusively fed from acetyl-CoA (1a). In this case, the spent C4 acids such as oxaloacetate also have to be replenished by reactions relying on acetyl-CoA. Hence, 1/3 of the carbon is lost into CO_2_ at pyruvate decarboxylase step, which is why a minimum of 1.5 mol of glucose will then be needed for producing 1 mol of itaconic acid, resulting in a maximum yield of 0.67 mol/mol _glucose_. The respiratory quotient for this pathway would be 0.89. Pathway **c**: The RQ for respiration biomass formation can be considered to be 1. Depending on the share of substrate used for respiration or biomass formation, the itaconic acid yield can be anywhere between 0 and 1 mol/mol _glucose_ while the corresponding respiratory quotient would be between 1 and 0.67, respectively. However, when CaCO_3_ is used in the medium, the itaconic acid will react to calcium itaconate once exported outside the cell, which releases an additional mol of CO_2_ (7). This leads to an apparent RQ between 1 and 1.33 for pathways **a**–**c**, depending on the itaconic acid production yield. The apparent quotient when using CaCO_3_ is shown in blue letters.

The biosynthesis of itaconic acid in the filamentous fungus *Aspergillus terreus* is directly derived from the Krebs cycle, where it is produced from *cis*-aconitate by decarboxylation via its key biosynthetic enzyme *cis*-aconitate decarboxylase (Fig. 1) [7]. The yeast *Ustilago maydis* in contrast, first isomerizes the *cis*-aconitate to *trans*-aconitate before decarboxylation [8]. Depending on the production yield and the involved metabolic reactions, the biosynthesis of itaconic acid has different implications on the respiratory quotient, which can be exploited to analyze its production non-invasively via offgas measurements, as described in Fig. 1.

Currently, industrial itaconic acid production is exclusively realized by a fermentation process using the filamentous fungus *Aspergillus terreus* [9]. However, itaconic acid production with *A. terreus* is a challenging process that is only efficient if certain prerequisites are fulfilled. These prerequisites are a high initial sugar concentration, a low fermentation pH, a strict manganese deficiency and a non-interrupted oxygen supply [10–12]. These prerequisites have been found especially difficult to be fulfilled in the context of cellulose-based itaconic acid production [13, 14]:

The bioconversion of cellulose into itaconic acid as well as other bio-commodities generally involves a hydrolysis of the cellulose into soluble sugars followed by a fermentation of the released sugars into the targeted product. This process configuration is called separate hydrolysis and fermentation (SHF). Recent attempts to produce itaconic acid in an SHF approach failed mainly because of manganese and other impurities contained in both the cellulose feedstock as well as in cellulase enzyme preparations used for hydrolysis [15]. Until now, production of cellulosic itaconic acid had been only proven successful after extensive purification of the cellulose hydrolysate using ion exchangers or activated charcoal [16–18], which impacts the economy of the process. Table 1 shows a summary of all known attempts on cellulose-based itaconic acid production available from literature. A strategy that has the potential to substantially increase the process economy is simultaneous saccharification and fermentation (SSF). This concept has the great advantage that the inherent inhibition of the cellulases by their hydrolysis products is completely avoided, since the released sugars are constantly consumed during the process. However, while sugars should not accumulate during SSF to prevent product inhibition of cellulases, high sugar concentrations are a prerequisite for efficient itaconic acid production using *A. terreus* [10, 13, 15, 19, 20]. Therefore, this process configuration is not suitable with *A. terreus* as a biocatalyst [15]. Another obstacle for an *A. terreus*-based SSF is the required low fermentation pH of 3.4–1.8, which is incompatible with the pH activity profile of conventional cellulases (pH 4–6.5).

**Table 1:**
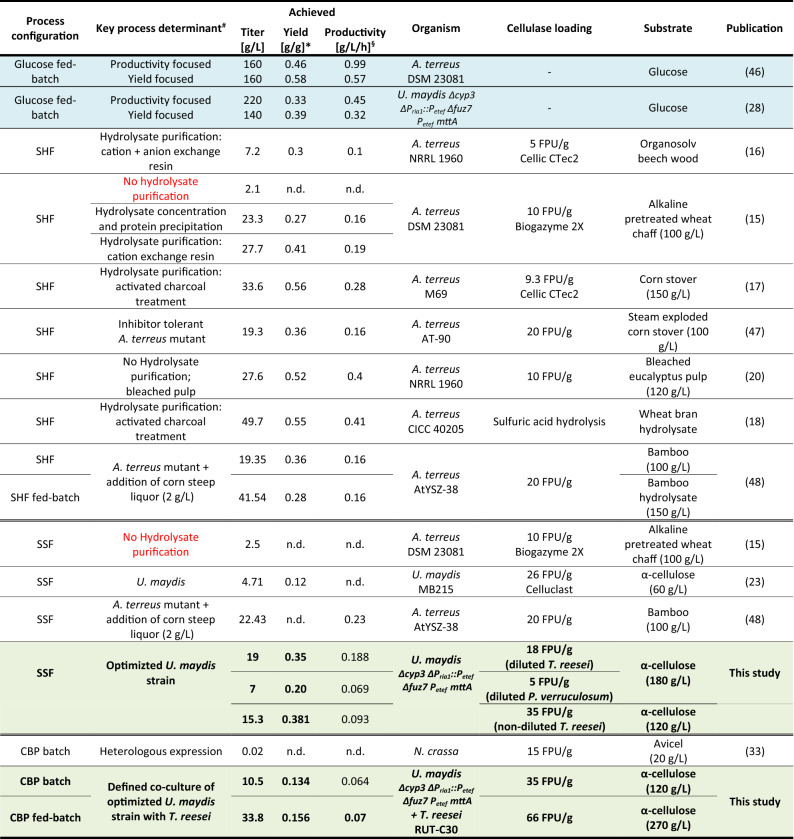
Attempts for cellulose-based itaconic acid production in literature

^#^The key determinant to overcome the challenges associated with cellulose-based itaconic acid production. For reference, cases without specific adaptions are shown in red. *Yield is based on consumed glucose equivalents excluding cellulase production phase. ^§^Averaged productivity excluding cellulase production phase. Glucose based reference datasets are shaded blue, results from this study are shaded green

*Ustilago maydis* is a promising alternative itaconic acid producer, which is capable of producing itaconate at a more neutral pH range (4.5–6.5) and with lower initial sugar concentrations. Furthermore, *U. maydis* is completely insensitive to manganese, which renders it much more suitable for the conversion of non-purified cellulose feedstocks. It has a single cell yeast morphology, which improves fermentation broth rheology and thereby facilitates efficient aeration in comparison to shear-sensitive fungi pellets [14, 21]. In addition, in terms of biosafety, *U. maydis* is classified as biosafety level 1 and can be even consumed as a food delicacy while *A. terreus* is an opportunistic human pathogen and has recently been classified into biosafety level 2 in some countries, which considerably restricts its use [22].

*U. maydis* has already proven suitable for SSF-based itaconic acid production, however reaching only very low yields despite application of high cellulase loadings of 0.1 g_Protein_/g_Cellulose_ or 26 filter paper units (FPU)/g_Cellulose_ [23]. Recently, itaconic acid production with *U. maydis* and other Ustilaginaceae such as *U. cynodontis* has been improved considerably by genetic engineering [24–27]. This, along with advances in bioprocess design enabled by the yeast morphology [27–29], has considerably enhanced the yield, titer, and rate of itaconic acid production by *Ustilago*.

A yet unachieved goal is the direct conversion of cellulose into itaconic acid without application of externally produced enzymes in a fully consolidated bioprocess (CBP). Although both *A. terreus* and *U. maydis* naturally possess biomass-hydrolysing enzymes [30–32], neither of the organisms is known to produce itaconic acid when grown on cellulose. Furthermore, their cellulase activity is far too low for efficient itaconic acid production from cellulose. To realize a CBP, different strategies are possible. One approach that has been realized recently is the genetic modification of a native cellulolytic organism like *Neurospora crassa* to enable the synthesis of itaconic acid. However, only 0.02 g/L of itaconic acid were produced from 20 g/L corn stover [33]. In general, the recombinant production of itaconic acid in non-native hosts has been proven challenging. Even the conversion of the citric acid producer *A. niger*, which is closely related to *A. terreus*, into an effective itaconic acid producer seems to be difficult [34–36]. Alternatively, a native itaconic acid producer could be engineered to produce carbohydrate-active enzymes, either by activation of dormant native genes [37, 38], or by heterologous expression [39]. However, this is no straightforward task as it often involves the high-level expression and secretion of multiple enzymes [40, 41]. A third strategy, which has the potential to unite both the benefits of native cellulase producers, and the high production capability of dedicated organisms for the target product synthesis, is co-culture fermentation. Due to synergistic effects of labor division, the performance of a co-culture can even be greater than the sum of the individual sub processes [42]. This strategy has already been proven successful for the production of different bio-commodities such as isobutanol, lactic acid, and fumaric acid [43–45]. Therefore, here we aim to establish a CBP by co-cultivating the hyper cellulolytic fungus *Trichoderma reesei* RUT-C30 (RFP1) with the engineered *U. maydis Δcyp3 ΔP*_*ria1*_*::P*_*etef*_* Δfuz7 P*_*etef*_* mttA* for itaconic acid production.

## Results and discussion

### Simultaneous saccharification and fermentation (SSF)

As a first step towards consolidated bioprocessing, itaconic acid production was assessed in a SSF setup to evaluate the capability of the engineered *U. maydis Δcyp3 ΔP*_*ria1*_*::P*_*etef*_* Δfuz7 P*_*etef*_* mttA* to produce itaconic acid under glucose-limiting conditions. Thereby, fermentation supernatants of two different cellulolytic fungi (*T. reesei* RUT-C30 (RFP1) and *Penicillium verruculosum* (M28-10) were compared as source for cellulases. These fungi have been found compatible with the presence of itaconic acid in a previous screening [49]. Since nitrogen limitation is a prerequisite for itaconic acid production with *U. maydis*, the residual NH_4_^+^-concentration present in the cellulase-containing supernatants had to be monitored [50]. The supernatants were diluted accordingly to reach a final NH_4_^+^-concentration equivalent to the 0.8 g/L NH_4_Cl, typically present in itaconic acid production medium for *U. maydis* [26, 28, 29]. This resulted in a final cellulase titer of 2.2 FPU/mL for the cultivations containing *T. reesei* supernatant and 0.6 FPU/mL for the cultivations containing *P. verruculosum* supernatant. These values corresponded to 18 and 5 FPU/g _cellulose_, respectively, and are close to the cellulase loading of 10 FPU/g _cellulose_, typically used for separate cellulose hydrolysis [51]. Since cellulose digestibility has a major impact on cellulose hydrolysis, 120 g/L of highly digestible Sigmacell as well as 120 g/L recalcitrant α-cellulose were tested as substrates [52]. All media were buffered to pH 6.5 using 100 mM MES buffer.

**Fig. 2:**
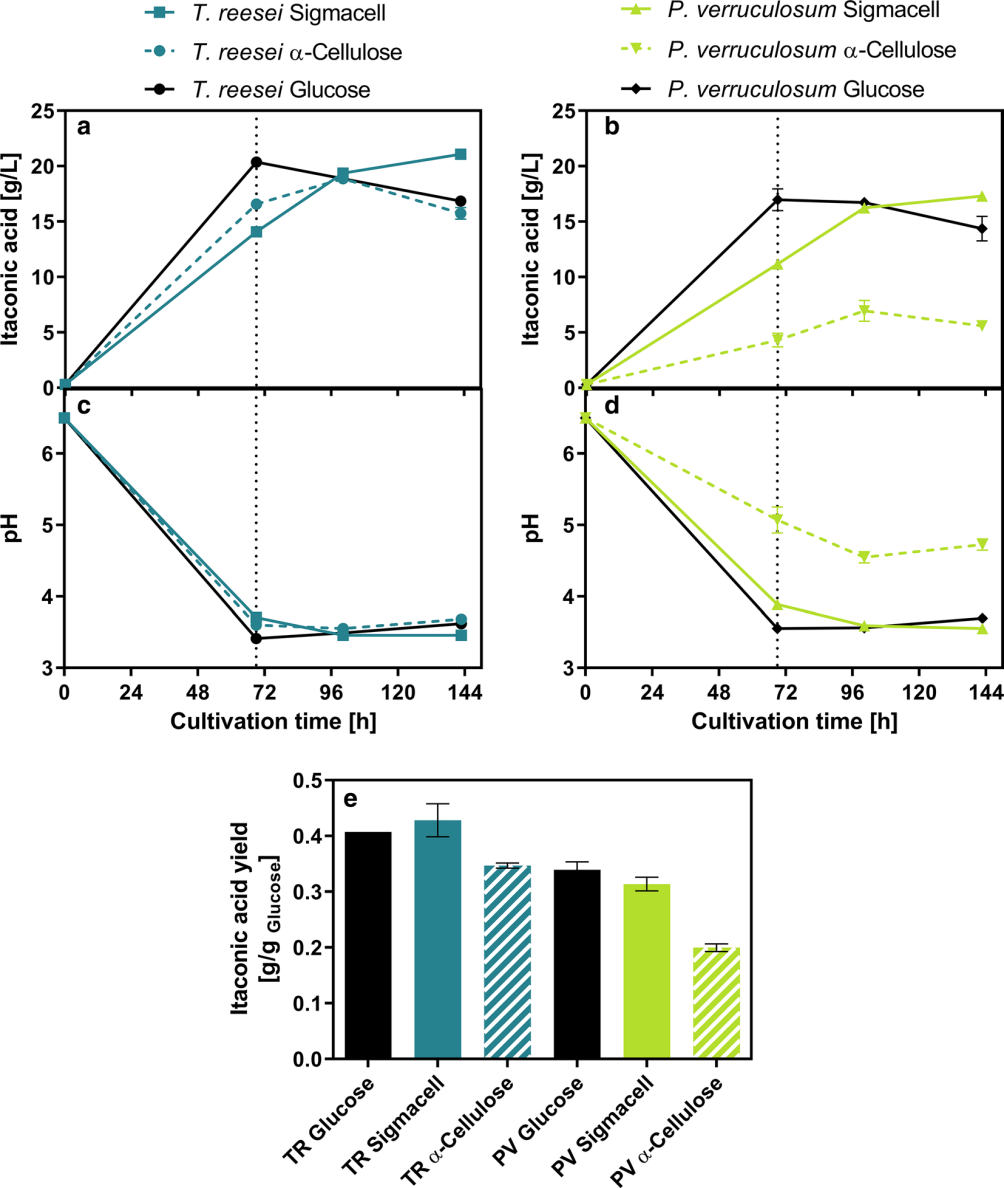
SSF-based itaconic acid production using *U. maydis Δcyp3 ΔP*_*ria1*_*::P*_*etef*_* Δfuz7 P*_*etef *_*mttA* with different cellulose substrates and sources of cellulases. **a** Shows the itaconic acid production during SSF using *T. reesei* (TR) enzymes (2.2 FPU/mL), **b** Shows the itaconic acid production during SSF using *P. verruculosum* (PV) enzymes (0.6 FPU/mL). **c**, **d** Show the corresponding pH profiles of the SSF cultures containing *T. reesei* or *P. verruculosum* enzymes, respectively. **e** Shows a comparison of the achieved itaconic acid production yields based on the consumed amount of glucose equivalents (1 g cellulose can yield 1.1 g glucose). Nitrogen-free itaconic acid production medium for *U. maydis* was supplemented with sterile filtered culture supernatants of *T. reesei* RUT-C30 (RFP1) or *P. verruculosum* M28-10. The residual NH_4_^+^ concentration in the culture supernatant was determined and the supernatants were diluted accordingly (1/5), so that the NH_4_^+^ transferred from the cellulase-containing supernatants is equivalent to the 0.8 g/L NH_4_Cl which is usually added to the medium as nitrogen source. Both culture supernatants were combined with either 120 g/L Sigmacell, 120 g/L α-cellulose or 50 g/L of glucose as a reference. The medium was buffered to pH 6.5 using 100 mM MES buffer. The cultures were inoculated to a final OD_600_ of 0.5 using a pre-culture of *U. maydis* Δ*cyp3* ΔP_ria1_::P_etef_ Δ*fuz7* P_etef_*mttA* with an OD_600_ of 18.2. The culture was performed with 25 mL filling volume in 250 mL Erlemeyer flasks at 200 rpm, 50 mm shaking diameter and 30 °C. Values shown are means of biological duplicates, error bars show deviation from the mean. The 100 h time point only shows single measurments from one replicate. The dotted line indicates the additional feeding of 60 g/L α-cellulose

As shown in Fig. 2, both *T. reesei* and *P. verruculosum* supernatants enabled itaconic acid production from cellulose by *U. maydis* during SSF. Using the *T. reesei* supernatant, similar itaconic acid concentrations as in a reference culture containing 50 g/L of glucose instead of cellulose were reached. The highest itaconic acid concentration achieved during the SSF was 21 g/L using Sigmacell as substrate, which is fourfold higher than previously demonstrated for a SSF using wildtype *U. maydis* MB215, and close to the values achieved with *A. terreus* (Table 1). Even using the relatively recalcitrant α-cellulose, a similar titer of 19 g/L itaconic acid was produced. Although the itaconic acid production using the *P. verruculosum* supernatant was generally lower than using *T. reesei* supernatant, especially with α-cellulose, the achieved titers were still considerable in relation to the almost fourfold lower cellulase activity of 0.6 FPU/mL. Remarkably, the itaconic acid yield based on the consumed amount of glucose equivalents from cellulose was essentially identical to the yield achieved using glucose in the reference and also comparable to yields achieved with *A. terreus* using purified cellulose hydrolysate (Table 1).

From the pH profile, it can be seen that in most of the cultures the pH dropped to about 3.5 after 69 h of cultivation. Since it is known that *U. maydis* stops itaconic acid production at such low pH values [53], it is possible that higher itaconic acid titers could have been achieved using a higher buffer concentration or active pH control, which would also have further improved the cellulose hydrolysis. This hypothesis was confirmed by comparing the itaconic acid production of glucose-supplemented SSF cultures either buffered with MES or with CaCO_3_ (Additional file 1: Fig. S1). In case of the MES-buffered culture, itaconic acid production stopped before the exhaustion of glucose when reaching pH 3.5. In contrast, the CaCO_3_-buffered culture that maintained pH between 6 and 7 continued itaconic acid production after exhaustion of the added glucose and further converted cellulose into itaconic acid. An added benefit of the CaCO_3_ addition is an in situ precipitation of the product as calcium itaconate, which alleviates product inhibition and facilitates downstream processing [28]. Calcium salt precipitation still belongs to the most mature and widely applied downstream technology for industrial organic acid production [28, 54].

### Consolidated bioprocessing (CBP) with co-cultures of *T. reesei* and *U. maydis*

**Fig. 3:**
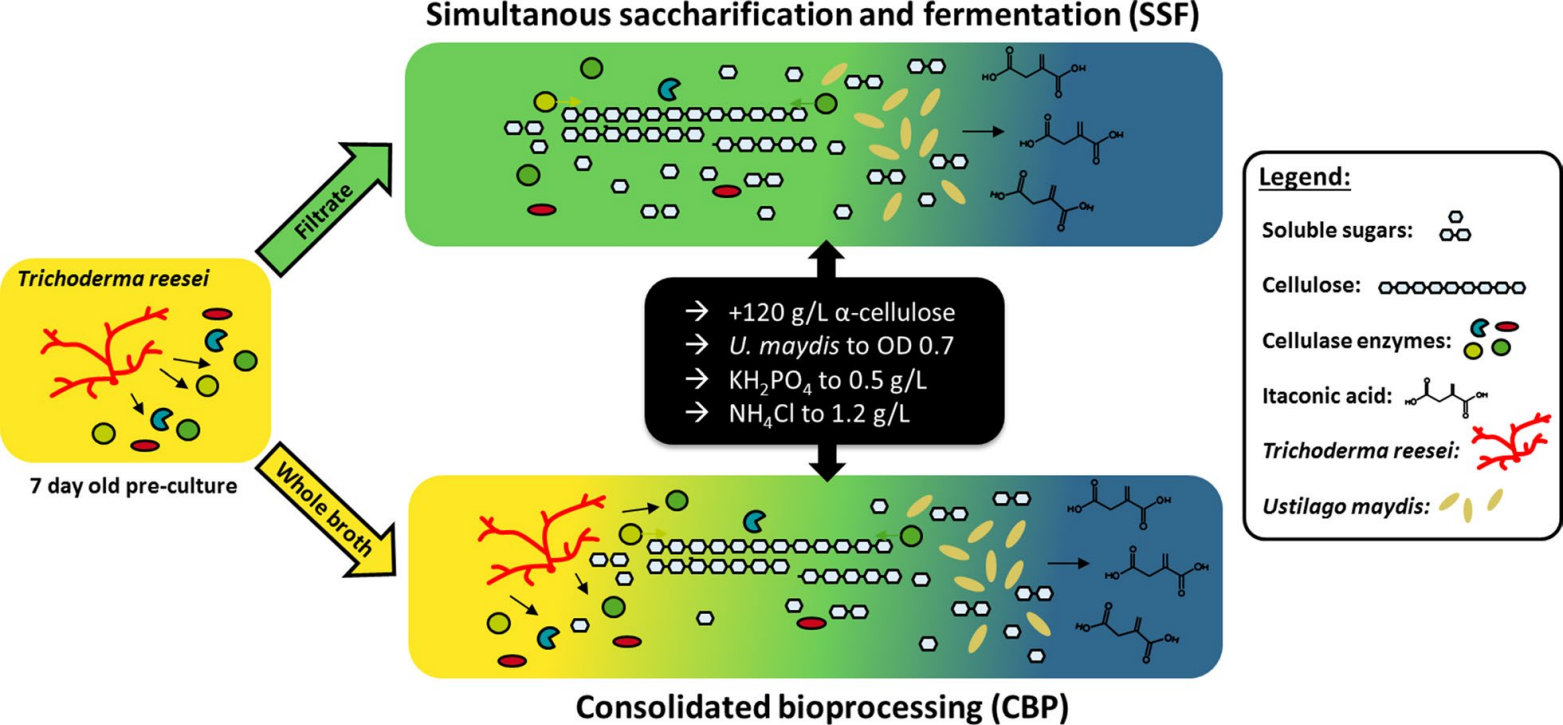
Experimental setup for comparison between CBP and SSF. *T. reesei* RUT-C30 (RFP1) cultures were grown for 1 week in co-culture medium buffered with 33 g/L CaCO_3_ to produce cellulases (shaded yellow). Thereafter, the cultures were pooled and phosphate and ammonium content of the culture was measured. Residual NH_4_^+^ was equivalent to 1.2 g/L NH_4_Cl and was not necessary to supplement before the inoculation of *U. maydis* since the NH_4_Cl concentration in itaconic acid production medium is only 0.8 g/L. Residual KH_2_PO_4_ was 0.18 g/L and was supplemented to a final concentration of 0.5 g/L to prevent preliminary phosphate limitation. For the SSF experiment, half of the pooled *T. reesei* culture broth was sterile filtrated and the filtrate distributed into three replicate Erlenmeyer flasks (25 mL each). For the CBP experiment, the pooled *T. reesei* culture broth was directly distributed to three replicate Erlenmeyer flasks (25 mL each). All cultures were supplemented with 120 g/L α-cellulose and 33 g/L CaCO_3_ and finally inoculated to OD 0.7 with *U. maydis* Δ*cyp3* ΔP_ria1_::P_etef_ Δ*fuz7* P_etef_* mttA.* While the SSF culture simutanously hydrolyses cellulose (shaded green) and produces itaconic acid (shaded blue) using the enzymes from the *T. reesei* pre-culture, the CBP culture is able to continue cellulase enzyme production in addition due to the presence of living T. reesei cells

As a next step, itaconic acid production was assessed in a CBP setup with a sequential co-culture of *U. maydis* and *T. reesei*. First, *T. reesei* was grown for 1 week in pure culture to produce sufficient cellulase enzymes, whereafter *U. maydis* was added to an OD of 0.67. To prevent a termination of itaconic acid production due to a decreasing pH, the medium was buffered with 33 g/L CaCO_3_. The performance of the CBP culture was directly compared to a corresponding SSF culture using undiluted sterile filtered supernatant of the same *T. reesei* pre-culture used for CBP. A schematic representation of the experimental setup is depicted in Fig. 3.

**Fig. 4:**
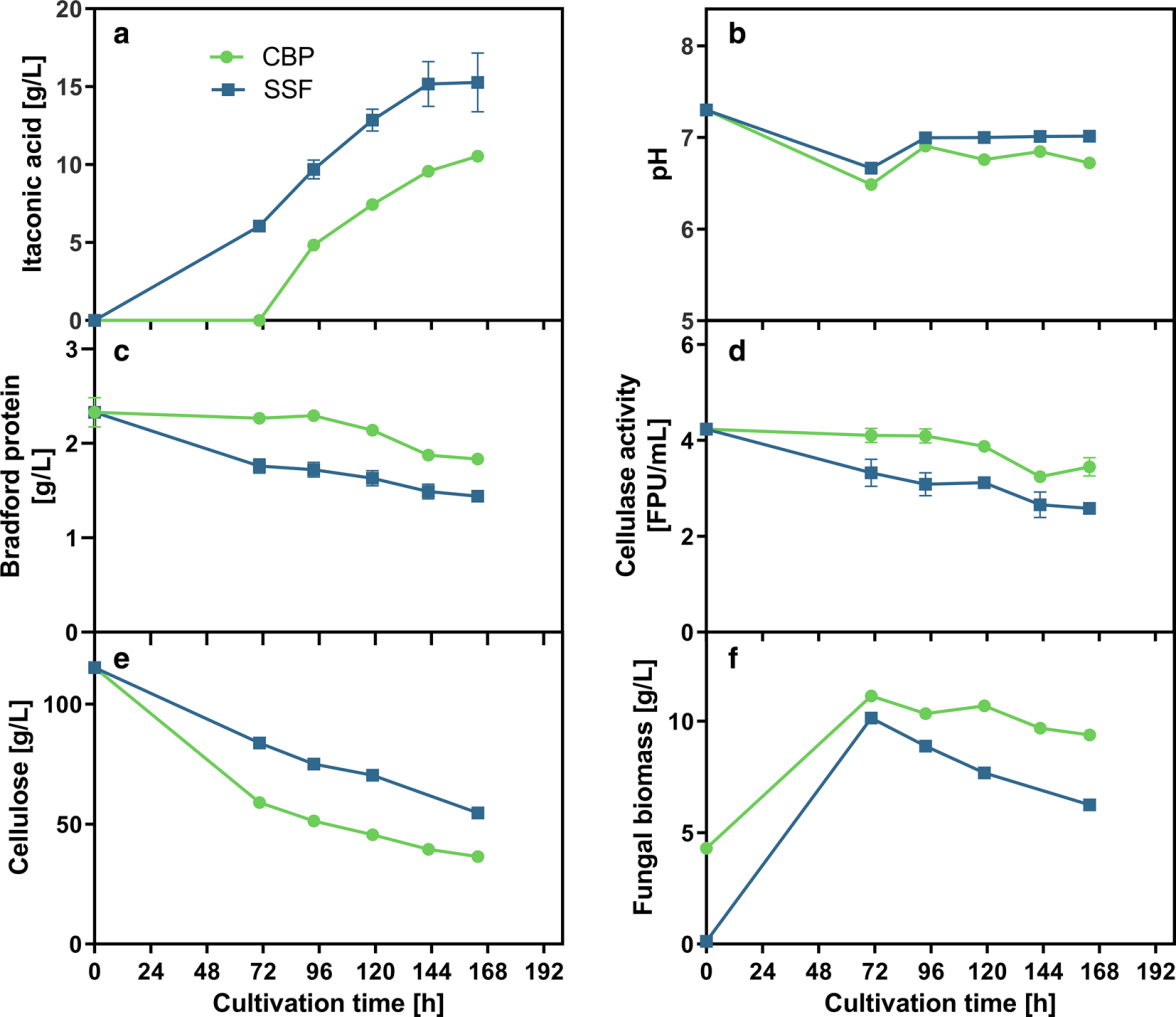
Comparison of CBP and SSF of cellulose to itaconic acid using *U. maydis* and *T. reesei*. **a** Shows the itaconic acid production of *U. maydis* Δ*cyp3* ΔP_ria1_::P_etef_ Δ*fuz7* P_etef_* mttA* from 120 g/L α-cellulose either using undiluted sterile-filtered *T. reesei* supernatant (SSF) or full culture broth (CBP) of a 1 week old pre-culture of *T. reesei* RUT-C30 (RFP1). **b** Shows the pH during cultivation. **c** Shows the protein concentration measured in the supernatant, which should correspond mainly to extracellular cellulases. **d** Shows the corresponding cellulase activity as measured by the filter paper assay. **e** Shows the residual cellulose concentration during the cultivation as determined by the Updegraff assay and **f** the corresponding dry weight of fungal biomass estimated from the weight loss during Updegraff assay. *T. reesei* RUT-C30 (RFP1) culture was grown for 1 week for cellulase production and then sterile filtered or directly used for the CBP experiment. The filtrate and the full culture broth were subsequently supplemented with 120 g/L α-cellulose, 0.32 g/L KH_2_PO_4_, 33 g/L CaCO_3_ and finally inoculated to a final OD_600_ of 0.67 using a pre-culture of *U. maydis* Δ*cyp3* ΔP_ria1_::P_etef_ Δ*fuz7* P_etef_*mttA*. The culture was performed with 25 mL filling volume in 250 mL Erlenmeyer flasks at 200 rpm, 50 mm shaking diameter and 30 °C. Values shown for itaconic acid and pH are means of biological triplicates, error bars show standard deviation. For cellulase activity, bradford protein, residual cellulose and fungal biomass, samples from the triplicates were pooled in order to collect sufficient sample volume and to make accurate gravimetric measurements and therefore only single values could be measured. For **c** error bars show standard deviation of technical triplicates, for **d** deviation of the mean from technical duplicates. Collected data are only shown until 164 h of cultivation. Beyond 164 h of cultivation, accurate and representative sampling was not possible anymore due to the high viscosity and inhomogeneity of the cultivation broth. The full dataset can be found in the Additional file 2: “Numerical data”

As can be seen in Fig. 4a, up to 10.5 g/L of itaconic acid was produced in the co-culture CBP. The SSF in contrast produced up to 15.3 g/L of itaconic acid. Thus, the CBP was clearly less effective in producing itaconic acid than the SSF. This could be already expected since two organisms have to share the same resources. The cellulose consumption (Fig. 4e) was clearly higher in the CBP compared to the SSF, especially during the initial 72 h growth phase. Since all conditions are identical between CBP and SSF except for the presence of living *T. reesei* cells, this increase in cellulose consumption can be clearly attributed to *T. reesei.* In turn, also the itaconic acid yield was affected by the CBP in comparison to the SSF. While a yield of 0.381 g/g _Glucose_ was reached in the SSF, only 0.134 g/g _Glucose_ were achieved in the CBP.

On the positive side, this increased cellulose consumption also demonstrates an enhanced cellulose hydrolysis performance with increased metabolic substrate demand. A recent publication on cellulosic malic acid production demonstrated that an increase in metabolic activity can drastically enhance cellulose hydrolysis without increasing cellulase concentration [55]. Therefore, the main challenge for optimizing the CBP setup is to channel the substrate consumption towards *U. maydis* and minimize the activity of *T. reesei* during the itaconic acid production phase. Still, some low residual activity of *T. reesei* could be beneficial, since the cellulase activity was more stable in the CBP compared to the SSF (Fig. 4c, d). This was likely due to constant re-synthesis of degraded cellulases by *T. reesei*.

**Fig. 5:**
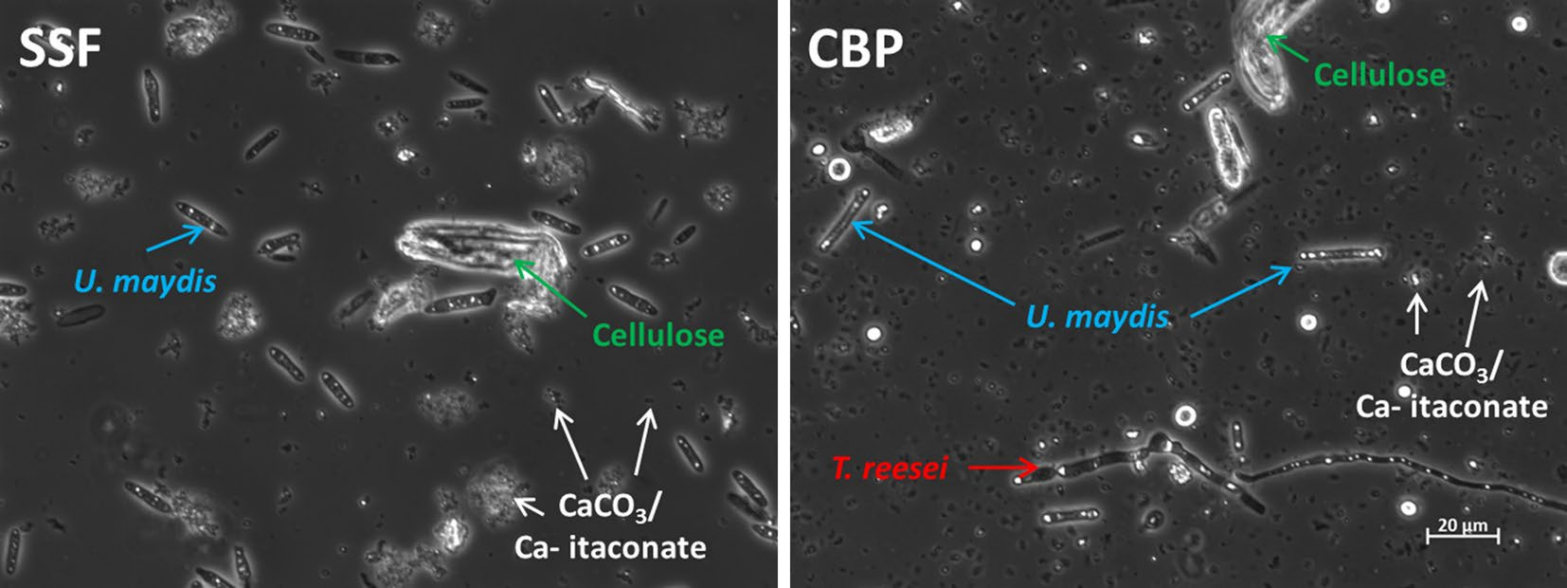
Representative phase contrast microscopic image of SSF and CBP samples acquired after 164 h of cultivation. Samples were diluted 1/10 in distilled water before microscopy (400X magnification). Small particles are CaCO_3_

The population dynamics between *U. maydis* and *T. reesei* were not investigated in detail. Still, microscopic examination of the samples suggested a relatively balanced population ratio between *T. reesei* and *U. maydis* towards the end of the culture. There were always cells visible from both *T. reesei* and *U. maydis* in randomly chosen fields of view (Fig. 5). The nitrogen supply in the cultures had to be limited in order to induce itaconic acid production by *U. maydis*. As a result of this limited nitrogen availability and competition with cellulase synthesis by *T. reesei*, both organisms could only grow until the shared nitrogen pool was exhausted. Hence, the *U. maydis* cell number per field of view was clearly lower in CBP compared to SSF, which might explain the lower itaconic acid productivity. If the nitrogen consumption of *T. reesei* in the CBP would have been compensated by appropriate addition of NH_4_^+^, similar *U. maydis* cell densities and itaconic acid productivities might have been reached as in the SSF.

The major benefit of CBP in contrast to SSF is that no enzymes had to be added and that cellulose can directly be converted into itaconic acid. Since the targeted substrates are cellulosic waste streams, which have very low cost, the production yield is less important. It has to be evaluated whether the economic benefits of the CBP process can compensate the yield loss. The outcome of this will most likely depend strongly on the price of cellulase enzyme production and cellulosic substrate.

### Influence of inoculation time

During the proof of principle CBP described above, *T. reesei* was cultured for one week in pure culture to produce sufficient cellulase enzymes before adding *U. maydis*. However, a one week pre-cultivation phase considerably lowers the average productivity of the co-culture compared to the SSF scenario (Table 2). To further optimize the co-culture and increase the productivity, the effect of inoculation delay between *T. reesei* and *U. maydis* on the CBP performance was studied. Four different additional inoculation delays were tested: 0 days (direct co-inoculation at the beginning), 3, 4, and 5 days. Because cellulase production by *T. reesei* usually starts after 2 days, it was expected that preliminary inoculation of *U. maydis* would strongly affect cellulase production due to competition for nitrogen. Therefore, 1 and 2 days delay were not tested. The experiment was performed in medium containing 5 g/L glucose for initial growth acceleration and 30 g/L α-cellulose. Furthermore, the experiment was carried out as fed-batch with regular feeding of α-cellulose powder for maximization of itaconic acid production.

**Table 2:** Comparison of itaconic acid productivity between SSF and CBP fermentations

	Total duration [h]	Itaconic acid titer [g/L]	Averaged productivity [g/L/h]	Source
SSF	164	15.3	0.093	Figure 4
CBP (itaconic acid phase only)	164	10.5	0.064	Figure 4
CBP (including cellulase production)	332	10.5	0.032	
CBP fed-batch (itaconic acid phase only)	478	33.8	0.071	Figure 6 [3 days delay]
CBP fed-batch (including cellulase production)	593	33.8	0.057	
Glucose fed-batch reference cultivation	87	42.2	0.485	Additional file 1: Figure S4

**Fig. 6:**
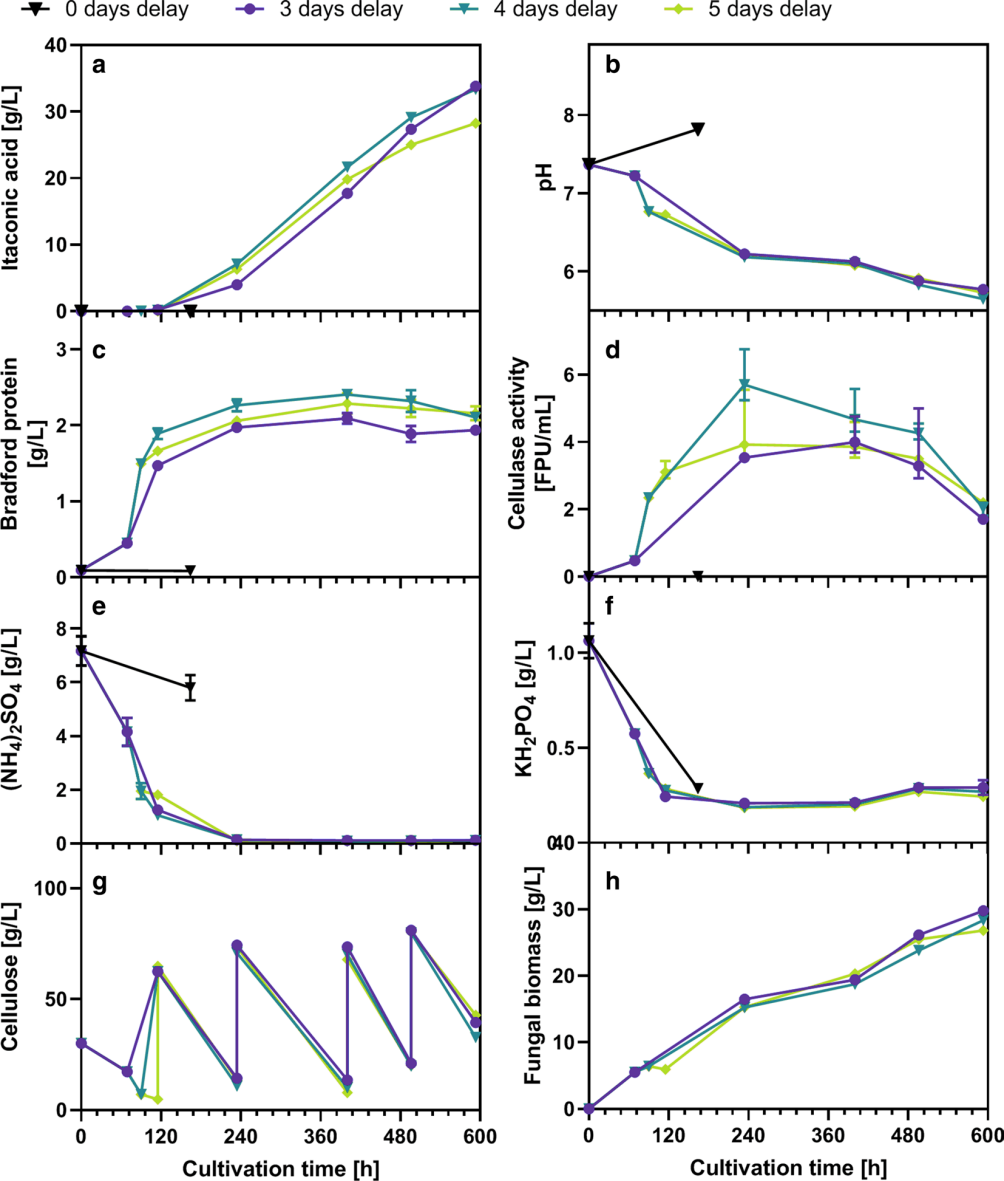
Investigation of the influence of inoculation delay on CBP performance. **a** Shows the itaconic acid production for different CBP cultivations initially inoculated with *T. reesei* RUT-C30 (RFP1), that where inoculated with *U. maydis* after different time delays ranging from 0 to 5 days delay. **b** Shows the pH during cultivation. **c** Shows the protein concentration measured in the supernatant, which should mainly correspond to extracellular cellulase enzyme, **d** Shows the corresponding cellulase activity as measured by the filter paper assay. **e** shows the (NH_4_)_2_SO_4_ concentration and **f** the KH_2_PO_4_-concentration during cultivation. **g** Shows the cellulose concentration during the cultivation as determined by the Updegraff assay and the increase in cellulose concentration after each feed. **h** Shows the corresponding dry weight of fungal biomass as estimated from the weight loss during Updegraff assay. The cultivation was performed starting from 30 g/L α-cellulose and 5 g/L glucose and was inoculated with 10^6^ spores/mL of *T. reesei* RUT-C30 (RFP1). The medium was buffered with 40 g/L CaCO_3_. *U. maydis* pre-culture was added after the indicated intervals to an OD of 1.14. All cultures were fed with 60 g/L α-cellulose as indicated in **g**. The cultivation was performed with 25 mL medium in 250 mL Erlenmeyer flasks at 200 rpm, 50 mm shaking diameter and 30 °C. Values show single measurements sampled from the same flask. Error bars show standard deviation or deviation from the mean from technical replicates: **a** n = 1; **b** n = 1; **c** n = 3; **d** n = 4 to 6; **e** n = 3; **f** n = 2; **g**, **h** n = 1. Until the inoculation of *U. maydis*, the 3, 4 and 5 days delay experiments can be considered as biological replicates. Because the first data point of each experiment was sampled just before the inoculation of *U. maydis*, the traces of the 4 and 5 days delay experiment were connected with the first values of the 3 and 4 days delay experiment, respectively. The full dataset can be found in the Additional file 2: “Numerical data”

When *T. reesei* and *U. maydis* were co-inoculated, the culture did not produce any cellulase enzymes nor any itaconic acid. Instead, exclusively *U. maydis* grew and consumed all glucose before *T. reesei* was able to germinate, thereby preventing the production of cellulases that would have enabled further growth of both organisms on cellulose. Because of the limited initial glucose concentration, a nitrogen limitation could not be reached, which explains the lack of itaconic acid production. Co-inoculation with vital cells of *T. reesei* instead of *T. reesei* spores might solve this problem since a starting quantity of cellulases would be present that would allow the co-culture to grow on cellulose instead of collapsing.

When *U. maydis* was added to the *T. reesei* culture after cellulase production had already started, itaconic acid production was successful. The influence of the different inoculation delays on both cellulase and itaconic acid production was surprisingly low (Fig. 6). Cellulase production of *T. reesei* is directly proportional to the available concentration of the nitrogen source. Because of this, it would have been expected that earlier inoculation of *U. maydis* would reduce cellulase production because of the competition for nitrogen. In this case, due to an earlier limitation of nitrogen also an earlier onset of itaconic acid production would have been expected. However, this was clearly not the case, suggesting only a very low growth and nitrogen consumption by *U. maydis*. To analyze the growth of *U. maydis*, a differential fluorescent staining technique was developed to clearly discriminate *U. maydis* cells from *T. reesei* and thereby allow for manual cell counting of the *U. maydis* population (see material and methods). For the cultures with 3 and 4 days inoculation delay, *U. maydis* only grew very slowly before the first cellulose feed. Starting from an initial inoculation density of 0.8∙10^7^ cells/mL (corresponding to a measured OD of 1.14), only a cell density of 2.7∙10^7^ and 1.7∙10^7^ cells/mL within 46 h and 25 h was reached, respectively, although the medium contained still all nutrients necessary for growth (Fig. 6e–g). The extent of growth inhibition becomes clearer in comparison to the growth of *U. maydis* in the instantly co-inoculated experiment. Here, *U. maydis* was able to grow to a cell density of 25∙10^7^ cells/mL in 24 h with just 5 g/L of glucose. For the experiments with inoculation delay, significant growth of *U. maydis* was only evident after increasing the carbon supply by feeding additional cellulose (Additional file 1: Figure S2). These phenomena may be explained by a higher affinity for sugars of *T. reesei* compared to *U. maydis*, enabling the former to out-compete the latter under sugar limitation. For reference, we observed µ_max_ values of 0.17 and 0.21 for *T. reesei* and *U. maydis,* respectively*,* when grown in pure culture at pH 7 in MES-buffered glucose media. Hence, under non-limited conditions *U. maydis* is the faster growing of both organisms. By feeding cellulose, the sugar concentration is increased, which enabled *U. maydis* to grow much faster.

Additionally, besides the temporal effect of the inoculation delay, there was also an unexpected viability effect that should have caused a growth benefit for *U. maydis* in the early inoculated experiments compared to the late inoculated experiments. The *U. maydis* inoculum was prepared from a YPD medium grown pre-culture that was washed twice with bi-distilled water and then stored as aqueous cell suspension at 0 °C for the different inoculation time points, so that the same stock could be used for all tested conditions. The viability of the aqueous inoculum was monitored for each inoculation time point by always inoculating a YPD medium filled flask in parallel to the CBP cultures and recording growth using online scattered light measurements. By observing an increase in the lag time with the age of the aqueous inoculum, it became clear that the viable cell density dropped significantly over time (Additional file 1: Figure S3). The increase in lag time by more than one doubling time (3.5 h) suggests at least a twofold difference in viable cell density. Despite the drop in viability, there was no impact on CBP performance. Thus, the sugar supply rate was the key factor determining population dynamics and sugar partitioning between *T. reesei* and *U. maydis,* while neither the inoculation delay nor inoculation density of *U. maydis* had a significant effect.

Also regarding itaconic acid productivity, the sugar supply rate (and thus, the cellulose hydrolysis rate) was most likely the limiting factor. For a glucose-based itaconic acid production reference, typically a cell density of 50∙10^7^ cells/mL and a productivity of 0.77 g/L/h are reached under the investigated conditions (Additional file 1: Figure S4). Since the determined *U. maydis* cell density during CBP ranged from 10∙10^7^ to 30∙10^7^ cells/mL, a theoretical itaconic acid production capacity of 0.16 to 0.46 g/L/h was present in the CBP. The fact that only a maximum productivity of 0.10 g/L/h was reached in the fed-batch CBP indicates that the cells were not producing itaconate at maximum capacity, likely due to the above-mentioned competition for glucose.

As envisaged, the total average productivity (including the cellulase production phase) in this fed-batch experiment was indeed higher than in the previous batch experiment with 7 day inoculation delay (Table 2). However, this effect was not related exclusively to the smaller inoculation delay or an earlier onset of itaconic acid production. Instead, the productivity was generally slightly higher and was sustained for a longer period, so that the influence of the cellulase production phase duration on the total average itaconic acid productivity was minimized. This was due to the regular feeding of cellulose and thus mainly a benefit of fed-batch fermentation in contrast to batch fermentation. The key factor controlling the start of itaconic acid production was the time point of the first cellulose feeding, which in all cases was synchronized to 5 days after start of the experiment and thereby likely also synchronized the itaconic acid production. This feeding regime was chosen because a preliminary mass feeding of cellulose would have compromised cellulase production by *T. reesei*. An earlier start of itaconic acid production could therefore be at the expense of cellulase activity.

### Detailed online process monitoring during co-culture CBP using online respiratory analysis

**Fig. 7:**
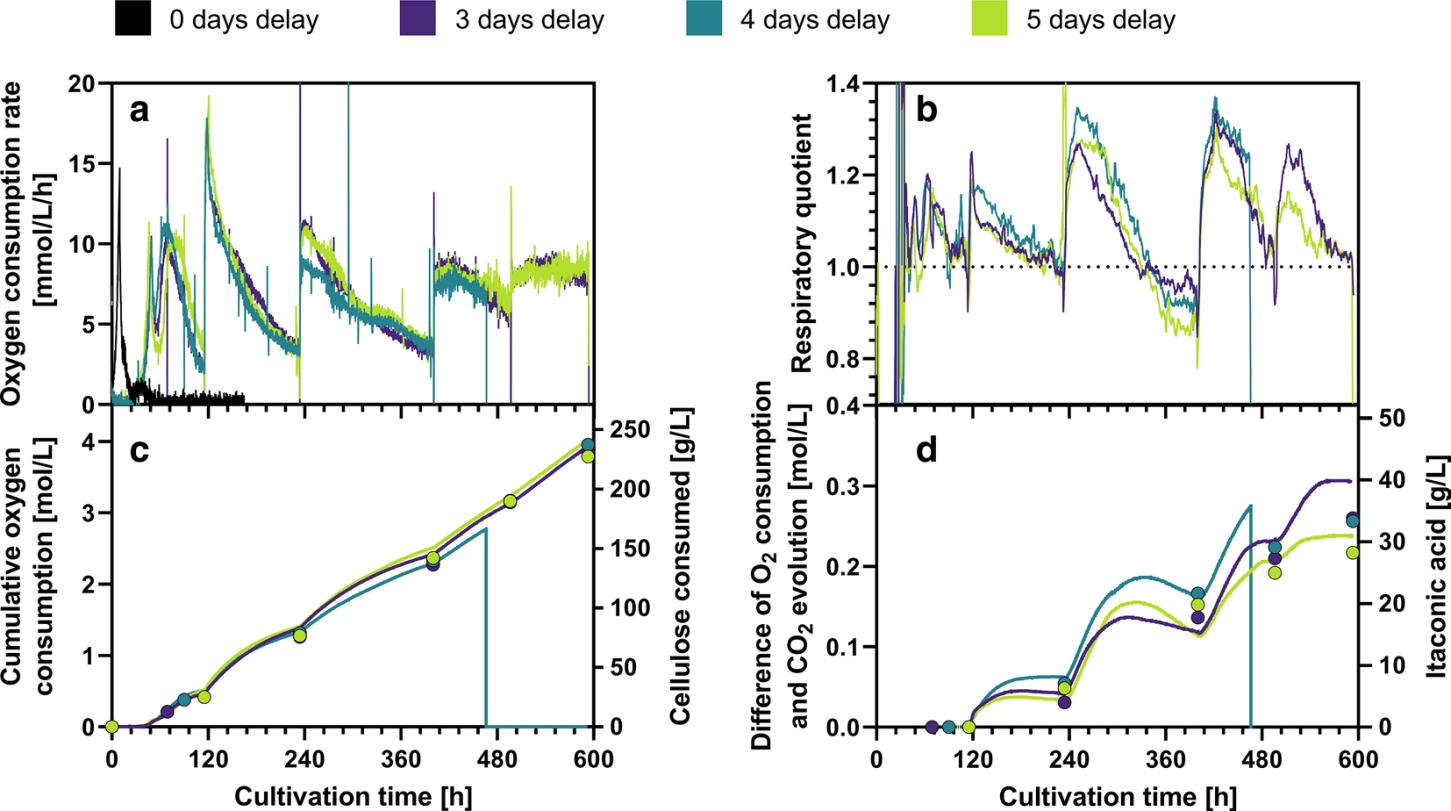
Respiration-based online process monitoring during co-culture CBP. **a** Shows the oxygen consumption rate. The first peak indicates the consumption of 5 g/L glucose, the second peak marks the consumption of the initial cellulose. The following peaks were caused by the cellulose feeding; a sharp increase in oxygen consumption was observed after every cellulose feeding interval. **b** Shows the corresponding respiratory quotient as a rolling average of the original data for clarity and noise reduction. **c** shows the cumulative oxygen consumption as an indicator for cellulose consumption. The closed circles show the actual cellulose consumption as determined from offline measurements. **d** Shows the difference between cumulative oxygen consumption and cumulative CO_2_ evolution as an indicator for itaconic acid formation. Closed circles indicate corresponding itaconic acid concentrations as determined from offline measurements. Y-axis were scaled in a way that 1 mmol CO_2_ evolution equals 1 mmol itaconic acid produced. Because of a software bug, the 4 days delay experiment stopped recording after 468 h. For comparison, values from a pure culture reference experiment of *U. maydis* grown in a glucose fed-batch can be found in Additional file 1: Figure S4

Up to now, consortium-based CBP has been proven successful only in academic research. One major obstacle for industrial application of such processes is a lack of suitable and established process control techniques. The substrate consumption for example is very difficult to assess in complex cultures containing solid cellulose particles. Here, the respiration rate of the fed-batch CBP was monitored online as a direct measure for microbial activity. As described in earlier studies [49, 56], cellulose consumption and thereby suitable time points for feeding fresh substrate could be estimated online based on the cumulative oxygen consumption. This way, intermittent starvation of the culture could be prevented (Fig. 7a, c).

The use of online respiration measurement further allowed to estimate the product formation online. Assuming the pure aerobic combustion of glucose as only carbon source for maintenance and biomass formation, the respiratory quotient can be considered to be 1 (Fig. 1, pathway C). During phases of significant formation of partially oxidized products such as itaconic acid, which is typically produced at a yield of 0.4 g/g in *U. maydis*, the RQ should be even below 1. Pure itaconic acid formation would cause an RQ of 0.67 (Fig. 1, pathway a). However, since CaCO_3_ was used for buffering of the cultures, the production of 1 mol itaconic acid would release an additional mole of CO_2_ by reacting with the carbonate, thereby increasing the RQ above 1 (Fig. 1, pathways a, b, blue values). This effect is depicted more clearly in Additional file 1: Figure S5, comparing the theoretical RQ in absence or in presence of CaCO_3_ in relation to the itaconic acid yield. Hence, an RQ above 1 in presence of CaCO_3_ indicates that itaconic acid production is ongoing, while a drop of RQ close to 1 indicates cessation of itaconic acid production. During the experiment, RQ values well above the theoretically expected values have been measured, because the used strain still produces considerable amounts of reduced by-products such as glycolipids, which result in an increased RQ [25, 57]. An explanation for this phenomenon is illustrated in Figure S5.

The profile of the RQ over time thereby serves as an indicator for the time-resolved itaconic acid yield during the process (Fig. 7b). While the global average itaconic acid yield was only 0.16 g/g, this averaged value likely results from dynamic fluctuations of phases with high yield (0.4 g/g) and phases without itaconic acid production. The data suggest that the actual itaconic acid yield was highly dependent on the cellulose concentration and thus sugar supply rate. After each feeding, the RQ went up to a maximum of about 1.3 before gradually decreasing to values close to or even below 1, where itaconic acid production likely stopped (Fig. 7b). When a surplus of substrate was available after a cellulose pulse, the yield approached values typically observed with glucose based fermentations, but then dropped as the substrate depleted. Thus, when the substrate supply was high, itaconic acid production was the dominant process, while respiration of *T. reesei* and *U. maydis* became more dominant with low substrate supply. This implies that a certain threshold substrate supply rate has to be sustained in order to enable itaconic acid production and that the maximum itaconic acid yield is only achieved under carbon non-limited conditions. This observation fits well to the observed influence of sugar supply rate on substrate partitioning and population dynamics between *T. reesei* and *U. maydis* observed at the early phase of the fermentation.

Assuming i) a baseline RQ of 1 for both *T. reesei* and *U. maydis*; ii) that any surplus CO_2_ is derived from acid reaction with CaCO_3_ and iii) that itaconic acid is the only acid formed, the amount of itaconic acid produced can be directly estimated from the difference between cumulated CO_2_ evolution and the cumulated O_2_ uptake. Figure 7d shows a comparison of itaconic acid production and online estimation based on respiration measurement. The axes are scaled in a way that 130 g/L itaconic acid equals 1 mol/L CO_2_ difference, hence 1 mol CO_2_ per mol itaconic acid (130 g/mol). Although the online estimation did not fit exactly to the HPLC measurements, the method can give an approximation and a clear trend of itaconic acid production. Underestimation can be explained by either production of reduced storage molecules, CO_2_ fixation or non-respirative oxidation reactions while over-estimation is most likely a result of other organic acid by-products, which have been observed in the CBP cultures but not during itaconic acid production from glucose (Additional file 1: Table S6). The online estimation suggests that the time-resolved itaconic productivity (the slopes in Fig. 7d) reached values up to 2.2 mmol/L/h (or 0.29 g/L/h) after each cellulose feeding, which implies that the actual itaconic acid production potential present in the CPB was considerably higher than the achieved averaged value of 0.07 g/L/h. These data suggest that if the duration of the feeding intervals of the cellulose are reduced, the space–time-yield could be increased up to fourfold, reducing the total process time to 240 h. The best way to optimize the process would be an RQ-controlled automatic feeding of cellulose powder.

### Techno-economic perspective

Nieder-Heitmann et al. performed a techno-economic analysis to compare sugar cane bagasse-based itaconic acid production via SHF to classical glucose-based production. Assuming an overall conversion yield of cellulose-rich sugarcane bagasse to itaconic acid between 0.086 and 0.188 g/g, a titer of 147 g/L and a productivity of 1.15 g/L/h, cellulosic itaconic acid would be already on-par or superior to glucose based itaconic acid [58]. While the calculated minimum selling price was most sensitive to yield and productivity, the titer was less important. It was shown that major economic burdens of the cellulosic SHF process were the high investment costs related to additional production facilities for separate hydrolysis and enzyme production as well as the energy requirement for the concentration of raw hydrolysates to the high sugar concentrations necessary for efficient itaconic acid production with *A. terreus*. Both of these major cost drivers were neutralized in the here presented SSF and CBP processes. Additional cost savings can be expected for the downstream processing as the itaconic acid precipitates in situ during fermentation, which makes product isolation less energy intense [54]. While the overall cellulose-to-itaconic acid conversion yield of 0.17 g/g in the fed-batch CBP already fits well into the economically viable scenario of Nieder-Heitmann et al., the productivity still needs to be significantly improved. However, a detailed techno-economic calculation for our case will be necessary to determine the minimum viable productivity, as cost savings from lower energy demand and lower investment costs will likely compensate to some degree for a lower productivity.

## Conclusion

The capability of *U. maydis* to produce itaconic acid under glucose-limiting conditions in an SSF process was verified, achieving a yield of 0.38 g/g using recalcitrant α-cellulose. This yield is the highest ever achieved in an SSF process, and it is comparable to that achieved on glucose (Table 1). The compatibility of *U. maydis* with a living *T. reesei* culture was evaluated in a sequential co-culture CBP and compared to an SSF process with undiluted *T. reesei* supernatant. Although the CBP process was inferior to the SSF configuration, a direct conversion of cellulose to a meaningful quantity of itaconic acid could be demonstrated for the first time. Improvement of substrate allocation to *U. maydis* will be a key strategy to maximize itaconic acid yield during CBP, with cellulose concentration being the main determining factor. This finding was further corroborated by online analysis of the metabolic processes during fed-batch CBP using online respiration measurements, which indicated that the time-resolved itaconic acid productivity and yield peaked shortly after feeding cellulose and dropped with the depletion of the cellulose. The proposed method for online process monitoring will be a valuable tool for future improvement of the process as the feeding regime can be adjusted precisely to maximize substrate availability and thereby minimize respiration. Another key variable that will be an obvious target for future research is the effect of nitrogen concentration, which ultimately limits cellulase formation as well as the cell density of *U. maydis* and might therefore significantly improve productivity. A remarkable itaconic acid titer of 33.8 g/L could be reached with the fed-batch CBP strategy, which is on par with current SHF based results (Table1). Overall, this study demonstrates the applicability of *U. maydis* for consolidated bioprocessing of cellulosic biomass in co-culture, thus further expanding the process window with this organism.

## Methods

### Microorganisms

*T. reesei* RUT-C30 (RFP1) (a red fluorescent protein-labeled clone [13] of the standard RUT-C30 strain obtained from American Type Culture Collection ATTC 56,765) was propagated at 30 °C on potato extract glucose agar medium (Roth, Karlsruhe, Germany) containing 40 mL/L of carrot juice (BIOBIO, Marken-Discount AG & Co. KG, Germany). *P.* *verruculosum* M28-10, kindly gifted by Dr. Gerhard Kerns (Saxon Institute for Applied Biotechnology, Leipzig, Germany), was propagated at 30 °C on medium containing 10 g/L malt extract (Difco; Becton, Dickinson and Company, USA), 40 mL/L carrot juice, 10 g/L wheat bran (Alnatura, Darmstadt, Germany), 10 g/L α-cellulose (Sigma-Aldrich, St. Louis, USA), 30 g/L agar (Difco; Becton, Dickinson and Company, USA). Spores were harvested from agar plates using 10 mL 0.01% (v/v) Tween 80 solution and washed twice with bi-distilled water. The spore concentration was determined in a Neubauer-Improved counting chamber (Superior Marienfeld, Lauda-Königshofen, Germany), adjusted to 10^9^ spores/mL and stored at 4 °C. This 1000 × concentrated stock was used for inoculation all experiments.

*U. maydis* Δ*cyp3* ΔP_ria1_::P_etef_ Δ*fuz7* P_etef_*mttA* is a genetically engineered variant of *U. maydis* MB215 with enhanced itaconate production, reduced by-product formation, and stabilized morphology [28]. This strain was propagated at 30 °C on yeast extract peptone dextrose (YPD) agar plates. Liquid overnight cultures of *U. maydis* were grown from single agar plate colonies at 30 °C in YPD medium. For inoculation of the experiments, YPD cultures were washed twice with bi-distilled water and used to inoculate the experiments to the final OD as indicated in the figure captions. For the initial SSF and batch CBP experiments, freshly prepared aqueous cell suspensions were used for inoculation. For the fed-batch CBP experiment, the same aqueous cell suspension stock was used for all tested inoculation delays and stored in an ice bath.

### Media and cultivation

All experiments were performed in 250-mL Erlenmeyer flasks with 25 mL filling volume at 30 °C, 200 rpm and 50 mm shaking diameter.

The itaconic acid production medium used in the initial SSF experiment was adopted from Geiser et al. [59]. Because the added cellulase-containing fermentation supernatants of the cellulase producers contained already residual NH_4_^+^, NH_4_Cl was omitted. The medium contained 0.2 g/L MgSO_4_·7H_2_O, 0.01 g/L FeSO_4_·7H_2_O, 0.5 g/L KH_2_PO_4_, 1 mL/L vitamin solution, 1 mL/L trace element solution, and as buffer 19.5 g/L (100 mM) 2-(N-morpholino) ethanesulfonic acid (MES) or CaCO_3_ as indicated in the figure captions. The pH of the MES stock solution was adjusted to 6.5 with 6 M NaOH solution. The trace element solution contained 15 g/L EDTA, 4.5 g/L ZnSO_4_·7H_2_O, 1 g/L MnCl_2_·4H_2_O, 0.3 g/L CoCl_2_·6H_2_O, 0.3 g/L CuSO_4_·5H_2_O, 0.4 g/L Na_2_MoO_4_·2H_2_O, 4.5 g/L CaCl_2_·2H_2_O, 3 g/L FeSO_4_·7H_2_O, 1 g/L H_3_BO_3_, and 0.1 g/L KI. The vitamin solution contained 0.05 g/L D-biotin, 1 g/L D-calcium pantothenate, 1 g/L nicotinic acid, 25 g/L myo-inositol, 1 g/L thiamine hydrochloride, 1 g/L pyridoxol hydrochloride and 0.2 g/L para-aminobenzoic acid. The medium was prepared as a 1.43 × concentrated stock solution. The solution was filtered through a 0.2 µm filter for sterilization. Before the experiment, the 1.43 × concentrate was diluted to its original concentration by addition of sterile bidest water and sterile filtered cellulase-containing fermentation supernatants. The cellulase-containing fermentation supernatants of *T. reesei* RUT-C30 (RFP1) and *P.* *verruculosum* M28-10 were produced in a stirred tank fermentation as described in Additional file 1: S7. The necessary amount of cellulose was directly weighted into empty Erlenmeyer flasks (3 g Sigmacell or 3 g α-cellulose) and steam-sterilized at 121 °C as powder before the liquid medium was added. Both types of cellulose were purchased from Sigma-Aldrich (St. Louis, USA). For the single feeding event during the SSF cultivation, 1.5 g the corresponding cellulose was steam-sterilized as powder and added separately to each Erlenmeyer flask.

The medium for the co-culture CBP was based on a medium published by Pakula et al., which was modified for co-culture compatibility with *A. terreus*, *U. maydis* and *T. reesei*, enabling both itaconic acid and cellulase production by the respective organisms [60, 61]. The final medium consisted of (NH_4_)_2_SO_4_ 7.6 g/L, KH_2_PO_4_ 0.8 g/L, MgSO_4_·7H_2_O 0.5 g/L, CaCl_2_·2H_2_O 0.23 g/L, NaCl 0.05 g/L, 5 g/L CaCO_3_, glucose 5 g/L, α-cellulose 30 g/L, peptone ex casein 2 g/L (N-Z-Amine® AS, Carl Roth, Karlsruhe, Germany), Tween 80 0.02% (v/v), trace element solution 2.5 mL/L. The main solution without trace elements and cellulose was always prepared as a 2 × concentrated stock solution that was set to pH 5.5 with 5 M NaOH. The solution was filtered through a 0.2 µm filter for sterilization. Before the experiment, the 2 × concentrate was diluted to its original concentration by addition of sterile bidest water and other supplementing solutions such as trace elements or glucose. The trace element solution (400 × concentrated) had the following composition: citric acid 180 g/L, Fe_2_(SO_4_)_3_ 2.29 g/L, ZnSO_4_·7H_2_O 16 g/L, CuSO_4_ 2.05 g/L, MnSO_4_·7H_2_O 1.6 g/L, H_3_BO_3_ 0.8 g/L, CoCl_2_·6H_2_O 2.71 g/L.

For the batch CBP experiment, *T. reesei* was cultivated at 200 rpm, 50 mm shaking diameter and 30 °C in three 1 L Erlenmeyer flasks with 100 mL filling volume for 7 days in the described co-culture medium buffered with 33 g/L CaCO_3_. Thereafter, the three cultures were pooled and phosphate and ammonium content of the culture was measured. Residual NH_4_^+^ was equivalent to 1.2 g/L NH_4_Cl and was not necessary to supplement before the inoculation of *U. maydis* since the NH_4_Cl concentration in itaconic acid production medium is only 0.8 g/L. Residual KH_2_PO_4_ was 0.18 g/L and was supplemented to a final concentration of 0.5 g/L to prevent preliminary phosphate limitation. For the CBP experiment, the *T. reesei* culture broth was distributed to three replicate Erlenmeyer flasks (25 mL each), for the corresponding SSF experiment, the residual *T. reesei* culture broth was sterile filtrated and also distributed into three replicate Erlenmeyer flasks (25 mL each). The necessary amount of cellulose and CaCO_3_ was directly weighted into the empty Erlenmeyer flasks and steam-sterilized at 121 °C as powder before the liquid was added. Thereby, the cultures were supplemented with 120 g/L α-cellulose and 33 g/L CaCO_3_.

For the fed-batch CBP, the KH_2_PO_4_ starting concentration in the co-culture medium was increased to 1.3 g/L to anticipate the KH_2_PO_4_ supplementation that was necessary in the batch CBP. CaCO_3_ was increased to 40 g/L.

The reference cultivation of *U. maydis* with 50 g/L glucose was performed in in the co-culture medium with only 1.5 g/L NH_4_SO_4_, 0.5 g/L KH_2_PO_4_, without cellulose and buffered with 40 g/L CaCO_3_.

### Sampling

1-mL samples were withdrawn from the same Erlenmeyer flasks during the cultivation (no sacrifice flasks). The weight of the Erlenmeyer flasks was determined before every sampling to correct the measured variables for evaporation. When necessary, bi-distilled water was added to compensate for significant evaporation.

### Analytics

Phosphate was determined according a method for orthophosphate determination published by the United states EPA [62]. Ammonium was determined according to a modified version of the Berthelot reaction [63]. The protein concentration of the culture supernatant was determined by a Bradford assay [64] using Coomassie Plus™ assay reagent (Thermo Scientific, Waltham, USA) and bovine serum albumin as standard. The measurement procedure was performed according to the manufacturer’s manual for microtiter plates. Cellulase activity in the culture supernatant was measured by the standard filter paper activity (FPA) assay according to the method of Ghose [65] adapted by Xiao [66]. The assay was performed in a 60-µL reaction volume in 96-well conical bottom PCR plates.

Soluble sugars and metabolites including glucose, cellobiose, xylose as well as itaconic acid, citric acid, malic acid and succinic acid were determined via HPLC analysis. To dissolve potentially precipitated calcium itaconate, the broth was diluted 6 × with 0.5 M HCl. After centrifugation of the fermentation samples (16,900* g*; 10 min; 4 °C) and a second centrifugation step of the resulting supernatant (3,000* g*; 10 min), the supernatant was analyzed by HPLC (Dionex HPLC UltiMate 3000, Thermo Scientific, Waltham, USA) at 65 °C using the following setup: Column: AMINEX Ion Exclusion HPX-87H, 300 × 7.8 mm (Bio-Rad Laboratories GmbH, Munich, Germany); detectors: Dionex™ Ultimate 3000 UV/VIS detector (Thermo Scientific, Waltham, USA) at 210 nm and RI-101 refractory index detector (Shodex, Munich, Germany); mobile phase: 5 mM sulfuric acid; flow rate: 0.7 mL/min.

For the standard determination of CDW in absence of cellulose particles, between 0.75 and 3 mL of sample were filled into pre-weighted conical bottom glass tubes and centrifuged (20 min, 3,000* g*, 4 °C). The supernatant was carefully pipetted off for other analytic procedures. The resulting pellet was washed two times by resuspension in 10 mL dest. water with subsequent centrifugation (20 min, 3000* g*, 4 °C) and careful removal of the water using a pipette connected via a collecting bottle to a vacuum pump. Thereafter, the washed pellet was dried overnight (at least 12 h) in a Speedvac device under vacuum at 40 °C and 300 g acceleration. Finally, the glass tube containing the dried pellet was weighed on a microbalance and the CDW was calculated by subtracting the empty weight of the tube.

For the determination of CDW and residual cellulose in samples containing cellulose particles, a modified version of the Updegraff method adapted by Ahamed & Vermette was used [67, 68]. First, the total dry weight of all solids (TDW) was determined as described for the standard CDW determination above. The resulting dry pellet was then re-suspended in 3 mL of Updegraff reagent and incubated for 30 min in a boiling water bath with a marble on top of the glass tube to reduce evaporation. Thereby, the Updegraff reagent selectively dissolves the fungal biomass, leaving the cellulose intact. Updegraff reagent is a mixture of 10 mL conc. nitric acid and 100 mL 80% acetic acid. After the incubation, the suspension was mixed with 3 mL 96% ethanol to facilitate sedimentation of the cellulose and centrifuged (20 min, 3000* g*, 4 °C). The resulting pellet was washed two times by resuspension in 10 mL dest. water with subsequent centrifugation (20 min, 3000* g*, 4 °C) and careful removal of the water using a pipette connected via a collecting bottle to a vacuum pump. Thereafter, the pellet was washed with 1 mL 70% ethanol without subsequent resuspension and dried overnight (at least 12 h) in a Speedvac device under vacuum at 40 °C and 300* g* acceleration. Finally, the glass tube containing the dried pellet was weighed on a microbalance and the weight of cellulose was calculated by subtracting the empty weight of the tube. The corresponding CDW was determined by subtracting the weight of cellulose from the TDW.

### Offgas analysis

The offgas analysis was realized using a commercial Transfer-Rate Online Measurement (TOM) system (Kuhner, Birsfelden, Switzerland) equipped with a mass flow controller.

### Online scattered light measurements

Online scattered light measurements were performed using the cell growth quantifier (CGQ) system (Aquila biolabs GmbH, Baesweiler, Germany).

### Microscopy and *U. maydis* cell counting

*T. reesei* and *U. maydis* were microscopically discriminated by their differing cell wall composition. Therefore, samples were stained with a mixture calcofluor, which predominantly stains β-1,4-glucan and trypan blue, which predominantly stains chitin [69]. The sample suspensions were first diluted 1/10 with bi-distilled water and then 1/10–1/20 with the following staining solution: 10 µg/mL trypan blue and 10 µg/mL calcofluor in 20 mM phosphate–citrate buffer pH 7.4. Finally, 11 µL of the diluted and stained suspension were pipetted into wells of a µ-Slide angiogenesis (ibidi GmbH, Gräfelfing, Germany) and analyzed at 10X magnification (Plan-Apochromat 10X objective with 1X tubelens optovar) by a ZEISS Axio Observer Z1 (Zeiss, Jena, Germany) inverted fluorescence microscope equipped with a Yokogawa CSU-X1 spinning disk unit. Calcofluor fluorescence was recorded with 405 nm laser excitation, RQFT 405/488/568/641 dichroitic mirror and BP 450/50 nm emission filter. Trypanblue fluorescence was recorded with 638 nm laser excitation, RQFT 405/488/568/641 dichroitic mirror and BP 690/50 nm emission filter. Per sample, a total of 8 different field of views were recorded. For extended focus depth, 5 slices covering a Z-dimension range of 32 µm were recorded and processed into a maximum projection image. Besides the differing morphology, *U. maydis* yeast cells showed stronger trypan blue fluorescence relative to *T. reesei* hyphae, which allowed manual counting of the yeast cells in the images.

## Supplementary information

**Additional file 1. **Supplementary materials.

**Additional file 2. **Numerical data.

## Data Availability

The data generated or analyzed during this study are included in this published article and its supplementary information files. Further datasets used and analyzed during the current study are available from the corresponding author on reasonable request.

## Abbreviations

CBP: Consolidated bioprocessing
CDW: Cell dry weight
CGQ: Cell growth quantifier
EPA: Environmental Protection Agency
FPU: Filter paper unit
HPLC: High-pressure liquid chromatography
IA: Itaconic acid
MES: 2-(*N*-Morpholino)ethanesulfonic acid
OD: Optical density at 600 nm
RFP: Red fluorescent protein
RQ: Respiratory quotient
SHF: Separate hydrolysis and fermentation
SSF: Simultaneous saccharification and fermentation
TDW: Total dry weight
TOM: Transfer-rate online measurement
YPD: Yeast extract peptone dextrose medium
