# Supplementary material for: Consolidated bioprocessing of cellulose to itaconic acid by a co-culture of Trichoderma reesei and Ustilago maydis
Source: Biotechnol Biofuels. 2020 Dec 14;13:207. doi: 10.1186/s13068-020-01835-4 (PMC7737373; doi:10.1186/s13068-020-01835-4)
Supplement: Supplementary file 1 — Additional file 1. Supplementary materials. [file 13068_2020_1835_MOESM1_ESM.pdf]

Supplementary Material

Consolidated bioprocessing of cellulose to itaconic acid by a co-culture of *Trichoderma reesei* and *Ustilago maydis*.

Ivan Schlembach[1, 2], Hamed Tehrani[3], Lars M. Blank[3], Jochen Büchs[4], Nick Wierckx[3,5], Lars Regestein[1], Miriam A. Rosenbaum[1, 2]*

* corresponding author

[1]Leibniz Institute for Natural Product Research and Infection Biology – Hans-Knöll-Institute, Jena, Germany

[2]Faculty of Biological Sciences, Friedrich-Schiller-University, Jena, Germany

[3]Institute of Applied Microbiology - iAMB, Aachen Biology and Biotechnology - ABBt, RWTH Aachen University, Germany

[4]AVT‑Biochemical Engineering, RWTH Aachen University, Germany

[5]Institute of Bio- and Geosciences IBG-1: Biotechnology, Forschungszentrum Jülich, Germany

Email addresses:

IS: ivan.schlembach@leibniz-hki.de

HT: hamed.tehrani@rwth-aachen.de

LB: lars.blank@rwth-aachen.de

JB: jochen.buechs@avt.rwth-aachen.de

NW: n.wierckx@fz-juelich.de

LR: lars.regestein@leibniz-hki.de

MR: miriam.rosenbaum@leibniz-hki.de

Supplementary Figures

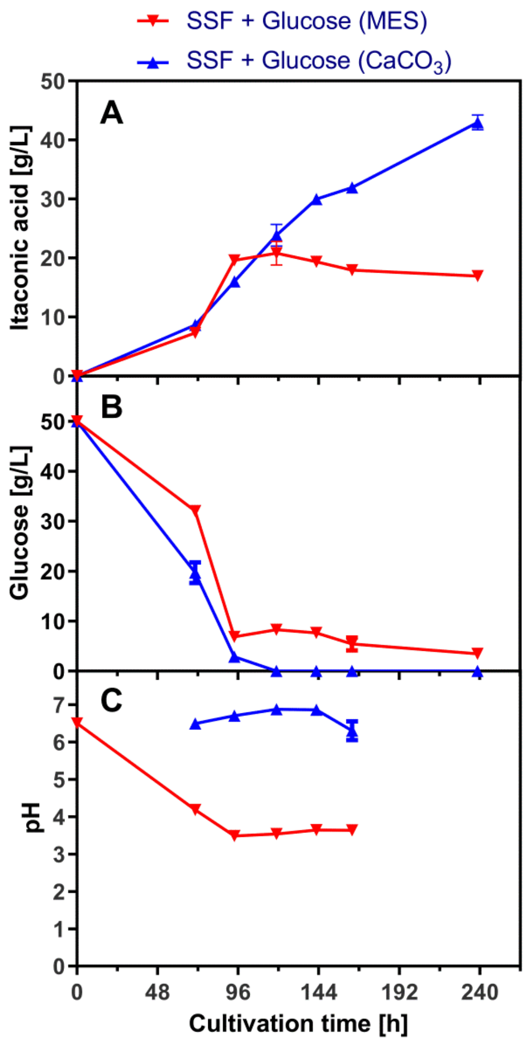

Figure S1: Comparison of itaconic acid production of glucose supplemented SSF cultures buffered with either 100 mM MES (red) or 33 g/L $CaCO_3$ (blue). (A) shows the itaconic acid production of *U. maydis* during SSF from 120 g/L α-cellulose using undiluted *T. reesei* supernatant (4.2 FPU/mL), supplemented with 50 g/L glucose. (B) shows the consumption of the supplemented glucose. (C) shows the corresponding pH profiles of the SSF cultures. *T. reesei* RUT-C30 (RFP1) culture was grown 1 week for cellulase production and then sterile filtered. The filtrate was subsequently supplemented with 120 g/L α-cellulose, 50 g/L glucose, 0.32 g/L KH_2PO_4 and finally inoculated to a final OD_{600} of 0.67 using a pre-culture of *U. maydis* Δ*cyp3* ΔP_{ria1}::P_{etef} Δ*fuz7* P_{etef}*mttA*. The cultivation was performed with 25 mL filling volume in 250 mL Erlenmeyer flasks at 200 rpm, 50 mm shaking diameter and 30 °C. Values shown are means of biological triplicates, error bars show standard deviation.

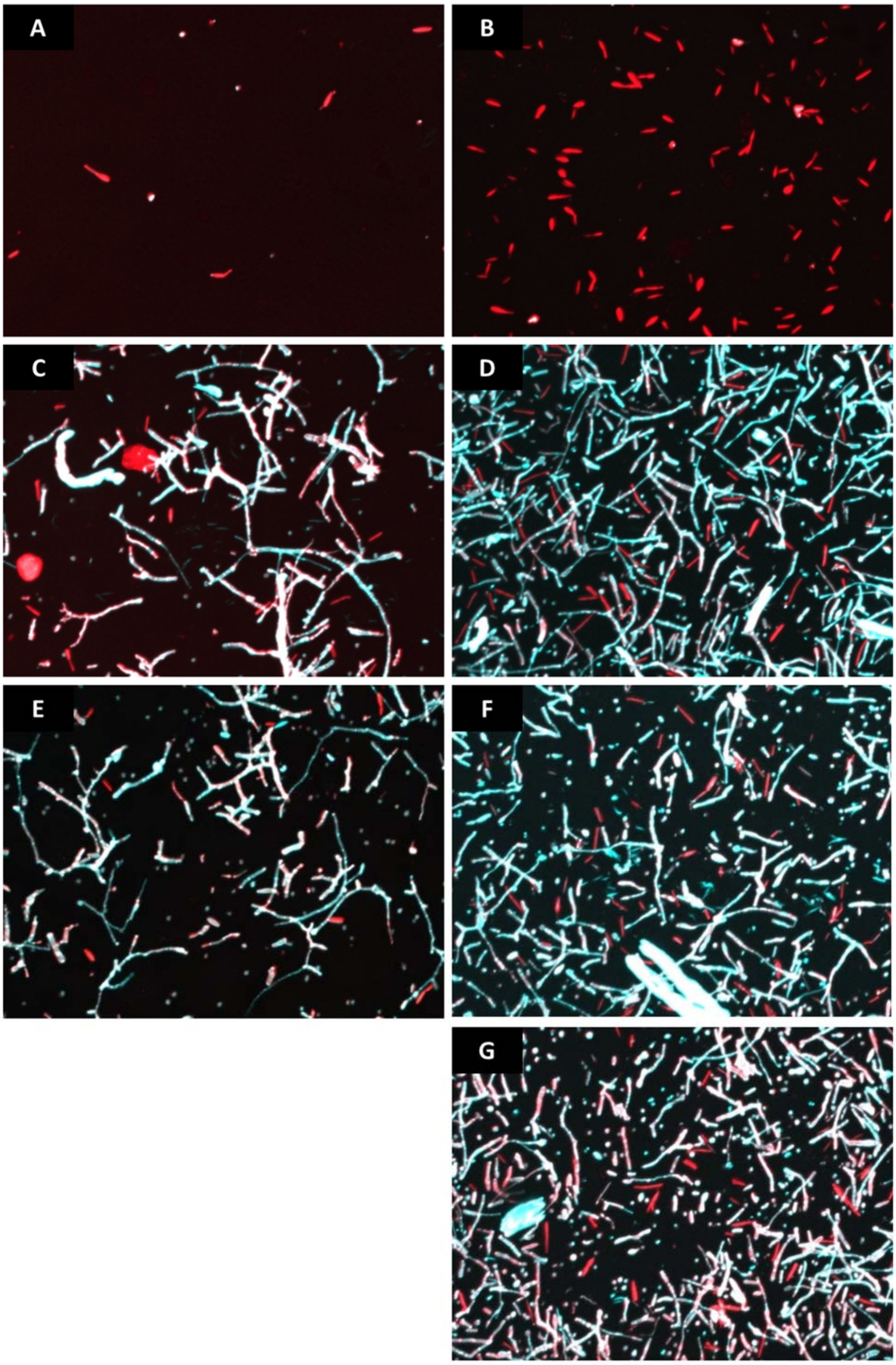

Figure S2: Microscopic estimation of *U. maydis* cell growth during fed-batch CBP before (C, E) and after the first cellulose feed (D, F, G). The samples were stained with a mixture of the fluorescent cell wall dyes calcofluor (cyan) and trypanblue (red) to differentiate *T. reesei* and *U. maydis*. Relative to *T. reesei*, *U. maydis* was more strongly stained by trypanblue and is therefore represented as red yeast cells while *T. reesei* hyphae appear cyan to white. (A) Shows a sample of the directly co-inoculated CBP experiment (0 days delay) at the start of the experiment. The picture is also representative for the starting *Ustilago* cell density in the other experiments. (B) shows the directly co-inoculated CBP experiment at the end of the cultivation (after 165 h). (C) and (E) show the *Ustilago* cell density of the 3 day and 4 day delay experiment, respectively, just before the first cellulose feed. (D), (F) and (G) show the *Ustilago* cell density of the 3,4 and 5 day delay experiment, respectively, at the end of the first feeding cycle (after 234h cultivation). Except of (A) that was diluted 1/100, all pictures show the microscopy of 1/200 diluted samples.

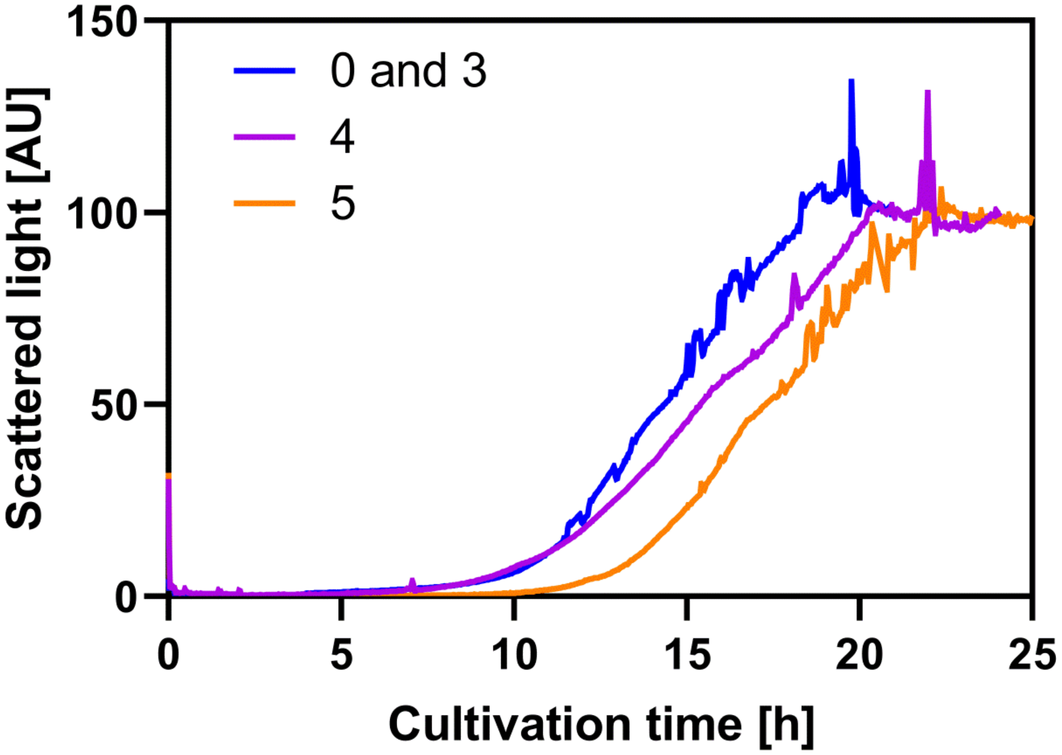

Figure S3: Viability check of *U. maydis* inoculum. The *U. maydis* inoculum was prepared from a YPD grown pre-culture, that was washed twice with bi-distilled water and then stored as aqueous cell suspension at 0°C for the different inoculation time points, so that the same stock could be used for all tested conditions. For each tested CBP inoculation time point, a YPD filled flask was inoculated in parallel and the growth was recorded using online scattered light measurements. The 0 and 3 day delay experiment were inoculated on the same date when the inoculum was fresh, the 4 days delay experiment 21h later and the 5 days experiment 46h later. While the growth rate was not largely affected by the age of the inoculum, the lag phase was increased by more than one doubling time (which is typically 3.5h) for the 5 day delay experiment.

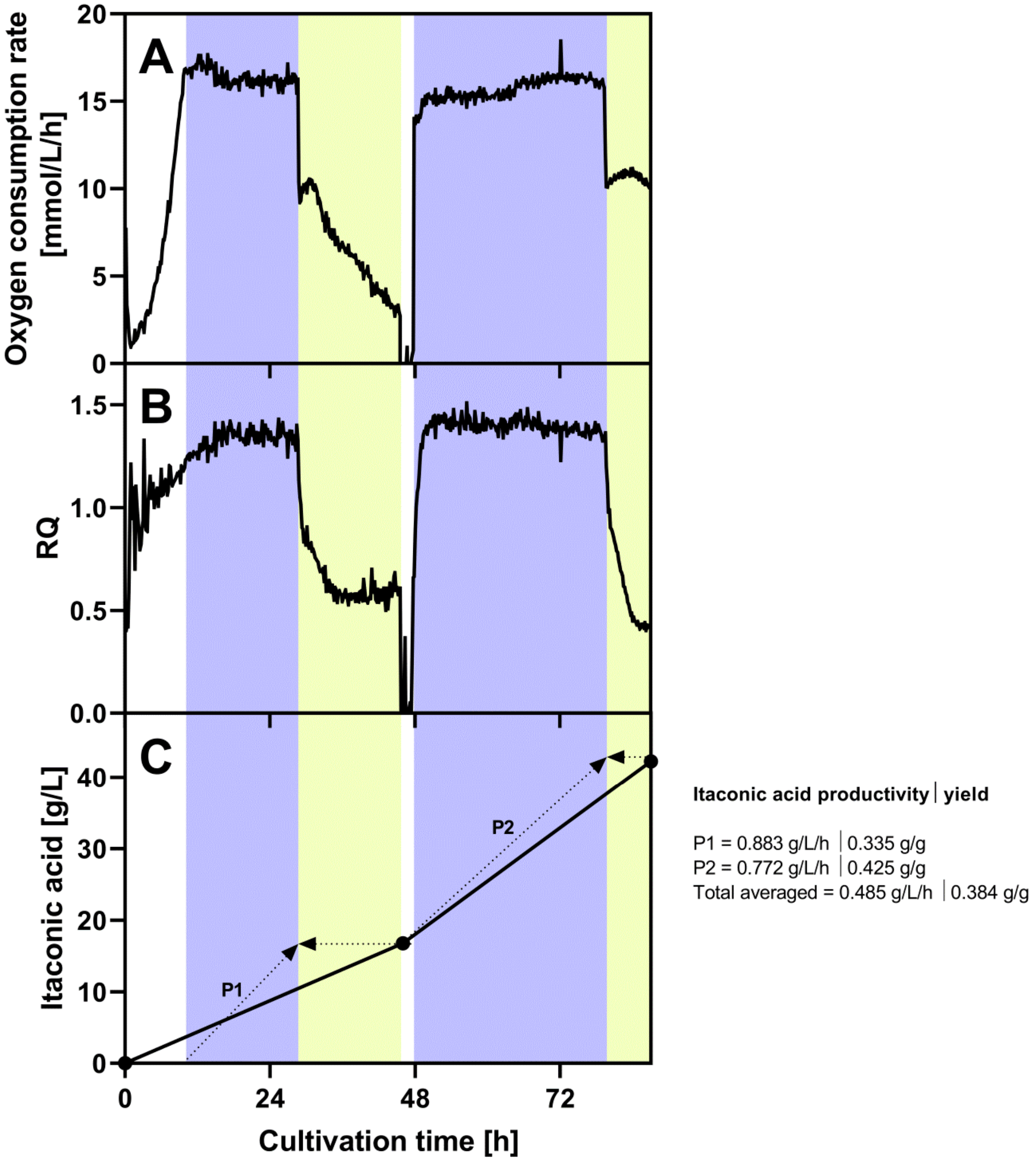

Figure S4: Reference data from a glucose based fed-batch cultivation of *U. maydis* *Δcyp3 ΔP$_{ria1}$::P$_{etef}$ Δfuz7 P$_{etef}$mttA*. (A) shows the oxygen consumption rate. The OTR increases until nitrogen limitation, then a plateau is reached. Thereafter, the culture enters the itaconic acid production phase, during which the RQ converges to 1.4 (blue shaded). Then, the OTR suddenly drops because the glucose is fully consumed. Subsequently, the OTR drops more gradually while the RQ (B) converges close to 0.5. During this phase, the organism likely consumes reduced storage compounds (green shaded). After feeding 60 g/L of glucose, the OTR increases back to the original plateau value and the RQ converges again to 1.4, entering another phase of itaconic acid production. After glucose is consumed, again, the organism switches to catabolism of storage compounds. (C) shows the itaconic acid production. The fermentation performance was evaluated for the different phases separately (P1 and P2) and also as a total average. For the calculation of the productivity, only actual duration of the itaconic acid production phase was considered, as marked by the dotted arrows. The initial glucose concentration was 50 g/L.

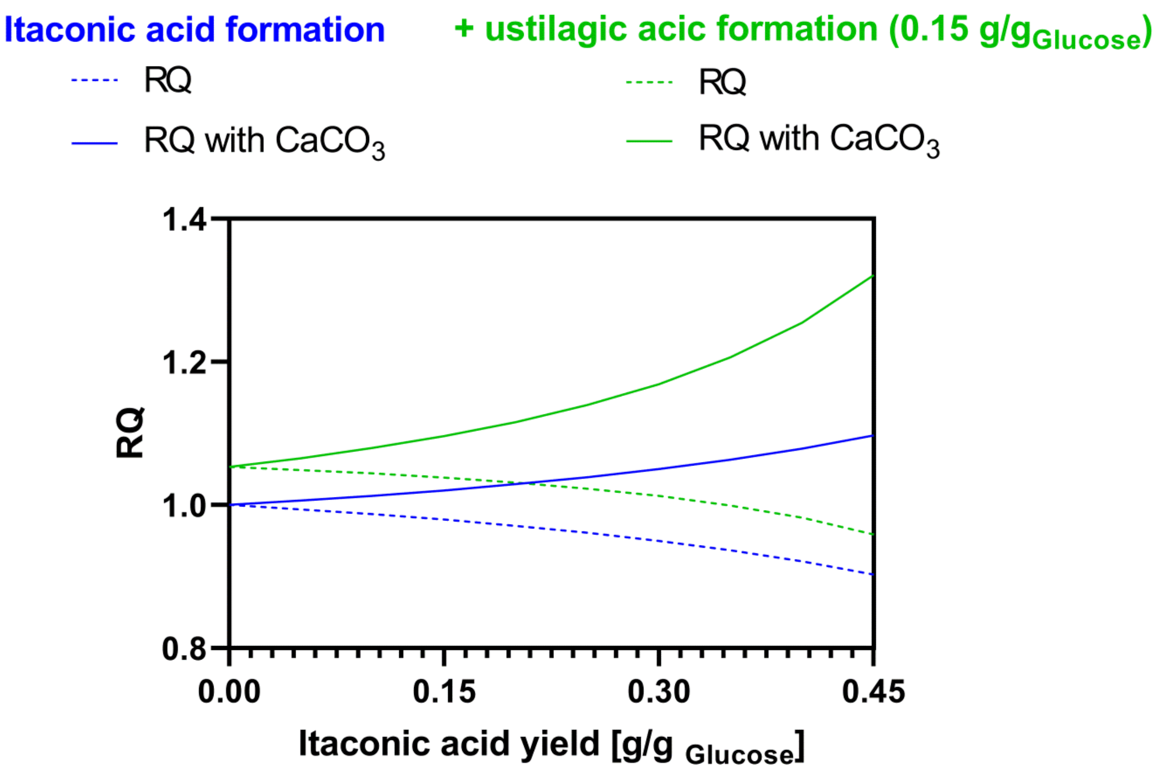

Figure S5: Expected RQ in relation to itaconic acid yield. The theoretical RQ was calculated based on reaction stoichiometry for different itaconic acid production yields from glucose. It was assumed that biomass formation was absent and itaconic acid formation and respiration are the only two metabolic processes occurring (dotted blue line). Since the fermentation broth was buffered with excess $CaCO_3$, every mol of itaconic acid produced releases an additional mol of CO_2 by reacting with the $CaCO_3$. This effect increases the RQ over 1 (solid blue line). With increase in itaconic acid yield the RQ rises up to a maximum of approx. 1.1 considering the maximum observed itaconic acid yield of *U. maydis* of 0.4 g/g. Since the RQ in the experiment rose close to 1.3 (Fig. 5B), itaconic acid production cannot fully explain the high RQ. However, it is known that *U. maydis* also produces other reduced by-products such as ustilagic acid, mannysolerythritol lipids (MEL) and others while producing itaconic acid. Assuming e.g., a parallel by-product formation at a yield of 0.15 g/g, an RQ of 1.3 is well possible. A scenario assuming ustilagic acid as the main by-product is shown in green lines. An itaconic acid yield increase by 0.08 g/g was recently determined when the by-product formation of ustilacic acid, MEL and triacylglycerol (TAG) were knocked out (1).

Table S6: Comparison of organic acid production of *U. maydis* production during fed-batch CBP and during a glucose based fed-batch fermentation.

	Itaconic acid [mmol/L]	Succinic/citric/malic acid [mmol/L]	Total acids [mmol/L]/ % itaconic acid
CBP 3 days delay	259	22/17/19	317/ **82%**
CBP 4 days delay	256	26/16/23	322/ **79%**
CBP 5 days delay	217	27/20/24	287/ **76%**
Glucose culture	325	0/0/0	325/ **100%**

Supplementary data S7: Stirred tank fermentation of *T. reesei* RUT-C30 (RFP1) and *P. verruculosum* M28-10 for the production of cellulase containing fermentation supernatants used in SSF.

T. reesei and *P. verruculosum* were cultivated in parallel in two Bioflo® 110 fermenters (New Brunswig Scientific, Edison, USA), each equipped with two six-blade Rushton impellers and a 1L glass fermentation vessel. Temperature control was realized by a combination of a heating mat wrapped around the glass vessel and a cold finger assembled into the vessel. Off-gas condensers and cold finger were connected to a cryostat. The head plate of the fermenter was equipped with a pH probe (Mettler-Toledo, Ohio, USA), a probe for dissolved oxygen (DO) (Hamilton, Höchst im Odenwald, Germany), a condenser for off-gas drying, a thermowell, an air sparger, a sampling port, ports for base and antifoam addition and a level sensor that was used for automatic antifoam addition. Antifoam 204 (Sigma-Aldrich, St. Louis, USA) was used as antifoam and was initially added at 0.5 mL/L to the medium. An air sparging tube with downward directed openings was chosen, because clogging was observed in spargers with upwards directed openings. The pH probe was calibrated before autoclavation using commercial pH standard solutions of pH 7 and pH 4 (Carl Roth, Karlsruhe, Germany) with the temperature probe of the fermenter placed within the calibration solution. Then the pH probe was assembled into the fermenter head plate and autoclaved together with the full assembly including the medium. The DO probe was always added afterwards into the autoclaved assembly and wiped with 70% ethanol for sterilization. The DO probe was then polarized overnight and finally calibrated at 30°C under vigorous air sparging and stirring (2 vvm and 800 rpm) for 100% dissolved oxygen tension (DOT) and by unplugging the cable and for 0% DOT.

The fermentation was performed in co-culture medium containing $(NH_4)_2SO_4$ 7.6 g/L, KH_2PO_4 0.8 g/L, $MgSO_4{\cdot}7H_2O$ 0.5 g/L, $CaCl_2{\cdot}2H_2O$ 0.23 g/L, NaCl 0.05 g/L, 5 g/L $CaCO_3$, glucose 5 g/L, α-cellulose 30 g/L, peptone ex casein 2 g/L (N-Z-Amine® AS , Carl Roth, Karlsruhe, Germany), urea 0.3 g/L, Tween 80 0.01% (v/v), trace element solution 2.5 mL/L. The fermenters were inoculated with 50 mL of 5 days old spore-inoculated precultures of either *T. reesei* RUT-C30 (RFP1) or *P. verruculosum* M28-10.

The initial culture conditions were: 0.5 L filling volume, aeration 1 vvm, agitator speed 400 - 800 rpm, agitation cascade to control DOT above 25%, 25 °C, pH control to 4.0 using 10% (v/v) NH_4OH. α-cellulose was fed intermittently every 5 h in charges of 2.4 g thrice a day, during night time the feeding was not performed. The cellulose feeding was realized by withdrawing 20 mL culture from the fermenter and replacing the withdrawn sample volume by injecting 20 mL of an autoclaved slurry of 120 g/L of α-cellulose suspended in co-culture medium. For this purpose, multiple 50 mL polypropylene syringes with custom bored wide openings were filled in advance with 2.4 g of α-cellulose and 20 mL of co-culture medium and then autoclaved. Towards the end of the cellulase production phase (189 h from the start of the fermentation), the pH control was shifted from 4.0 to 3.35. After 217 h from the start of the fermentation, 300 mL of culture broth was harvested from both fermenters and sterile filtered for the SSF experiment using *U. maydis*.

References:

1. Becker J, Hosseinpour Tehrani H, Gauert M, Mampel J, Blank LM, Wierckx N. An *Ustilago maydis* chassis for itaconic acid production without by-products. Microb Biotechnol. 2020;13(2):350-62.
